# A new specimen of *Prolacerta broomi* from the lower Fremouw Formation (Early Triassic) of Antarctica, its biogeographical implications and a taxonomic revision

**DOI:** 10.1038/s41598-018-36499-6

**Published:** 2018-12-20

**Authors:** Stephan N. F. Spiekman

**Affiliations:** Paläontologisches Institut und Museum der Universität Zürich, Karl-Schmid-Strasse 4, 8006 Zürich, Switzerland

## Abstract

*Prolacerta broomi* is an Early Triassic archosauromorph of particular importance to the early evolution of archosaurs. It is well known from many specimens from South Africa and a few relatively small specimens from Antarctica. Here, a new articulated specimen from the Fremouw Formation of Antarctica is described in detail. It represents the largest specimen of *Prolacerta* described to date with a nearly fully articulated and complete postcranium in addition to four skull elements. The study of this specimen and the re-evaluation of other *Prolacerta* specimens from both Antarctica and South Africa reveal several important new insights into its morphology, most notably regarding the premaxilla, manus, and pelvic girdle. Although well-preserved skull material from Antarctica is still lacking for *Prolacerta*, a detailed comparison of *Prolacerta* specimens from Antarctica and South Africa corroborates previous findings that there are no characters clearly distinguishing the specimens from these different regions and therefore the Antarctic material is assigned to *Prolacerta broomi*. The biogeographical implications of these new findings are discussed. Finally, some osteological characters for *Prolacerta* are revised and an updated diagnosis and phylogenetic analysis are provided.

## Introduction

*Prolacerta broomi* is a medium sized non-archosauriform archosauromorph with a generalized, “lizard-like” body type. Many specimens, mostly consisting of cranial remains, have been described and *Prolacerta* is considered one of the best represented early archosauromorphs^1–3^. *Prolacerta* is well known from the *Lystrosaurus* Assemblage Zone (AZ) of South Africa (Katberg Formation, Induan, Early Triassic)^4^, but specimens have also been reported from the lower Fremouw Formation of Antarctica (Induan)^5^. The taxon has been extensively discussed in relation to early diapsid evolution and was originally considered an intermediate form between “lizards” and basal diapsids such as *Youngina capensis*^6^. Later it was placed in the clade that was alternately called “Protorosauria”^7,8^ or “Prolacertiformes”^9^ that also included tanystropheids, drepanosaurs, and *Protorosaurus speneri*, as well as some lesser known taxa. This clade is now generally considered to be polyphyletic and recent analyses have retrieved *Prolacerta* as a very close relative to Archosauriformes^10–14^.

In his description of the first-discovered Antarctic *Prolacerta* material, Colbert described 17 new specimens^1^. Although he pointed out several small morphological differences, he considered these specimens to be conspecific with *Prolacerta broomi* specimens from South Africa. This identification supported the view at the time that the Antarctic fauna from the lower Fremouw Formation was nearly indistinguishable from that of the *Lystrosaurus* AZ of the Karroo Basin of South Africa. In fact, the discovery of many congeneric Early Triassic amniotes between the two regions (e.g. *Lystrosaurus*, *Procolophon*, and *Thrinaxodon*) provided an important line of evidence for the theory of continental drift^15–17^. Although some differences have since then been established^5,18^, both faunas are still considered very similar. Almost all authors that have addressed the osteology of *Prolacerta* following Colbert’s initial description, either focusing specifically on *Prolacerta*^3,19,20^ or considering it within a comparative and phylogenetic framework^13,21,22^, have only considered the South African material. The few studies considering the Antarctic material all referred to one Antarctic specimen, AMNH 9502 (American Museum of Natural History, USA), for their phylogenetic analyses^11,12,23^. Therefore the Antarctic material is currently considered to be poorly documented and understood^10^.

**Figure 1:**
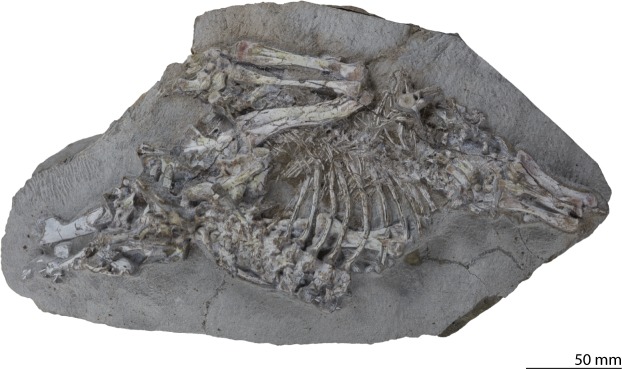
Specimen UWBM 95529.

The aim of this study is to present a full description of a newly discovered *Prolacerta* skeleton, UWBM 95529 (Burke Museum of Natural History and Culture, USA) that constitutes the most complete Antarctic specimen to date (Fig. 1) and to provide an osteological revision of *Prolacerta* with a focus on a detailed comparison between the Antarctic and South African specimens of the taxon. Additionally a number of new insights into the morphology, particularly of the postcranium, are presented and an emended diagnosis and updated phylogenetic analysis are provided.

## Systematic Paleontology

DIAPSIDA Osborn, 1903^24^

ARCHOSAUROMORPHA Von Huene, 1946^25^

PROLACERTA BROOMI Parrington, 1935^6^

### Emended diagnosis

*Prolacerta broomi* is a non-archosauriform archosauromorph distinguished from other archosauromorphs by the combined presence of the following characters: presence of septomaxillae; presence of recurved maxillary teeth; long horizontal dorsal margin of the maxilla with a concave posterior margin; well-developed posterolateral process on the frontal; two foramina for the passage of the carotid arteries and parasphenoid crests on the ventral surface of the parasphenoid; presence of medially directed teeth on the medial margin of the pterygoid; conical process on the proximal end of the humerus; ambiens process on the anterior margin of the pubis; ischial symphysis raised on a triangular shaped peduncle.

### Notes on previous diagnosis

The most recent diagnosis of *Prolacerta* was provided by Ezcurra^10^. It specified *Prolacerta broomi* as follows: “*Prolacerta broomi* is a basal archosauromorph distinguished from other saurians by the presence of: septomaxillae; notch on the ventral margin of the alveolar margin along the premaxilla-maxilla suture; conspicuous posterolateral exposure of the lacrimal duct openings; well-developed posterolateral process on the frontal, resulting in an acute-angled and V-shaped suture between frontals and parietals; parietals lacking a sub-rectangular fossa on the posterior half of the dorsal surface of the bones; absence of postparietals; extensive contact between the surangular and the prearticular in the articular region of the lower jaw.” The new interpretations based on new morphological information revealed by UWBM 95529, in particular with regards to the postcranium, as well as the re-evaluation of the both the South African and Antarctic *Prolacerta* material, merit a modification of this diagnosis.

### Comments

**Figure 2:**
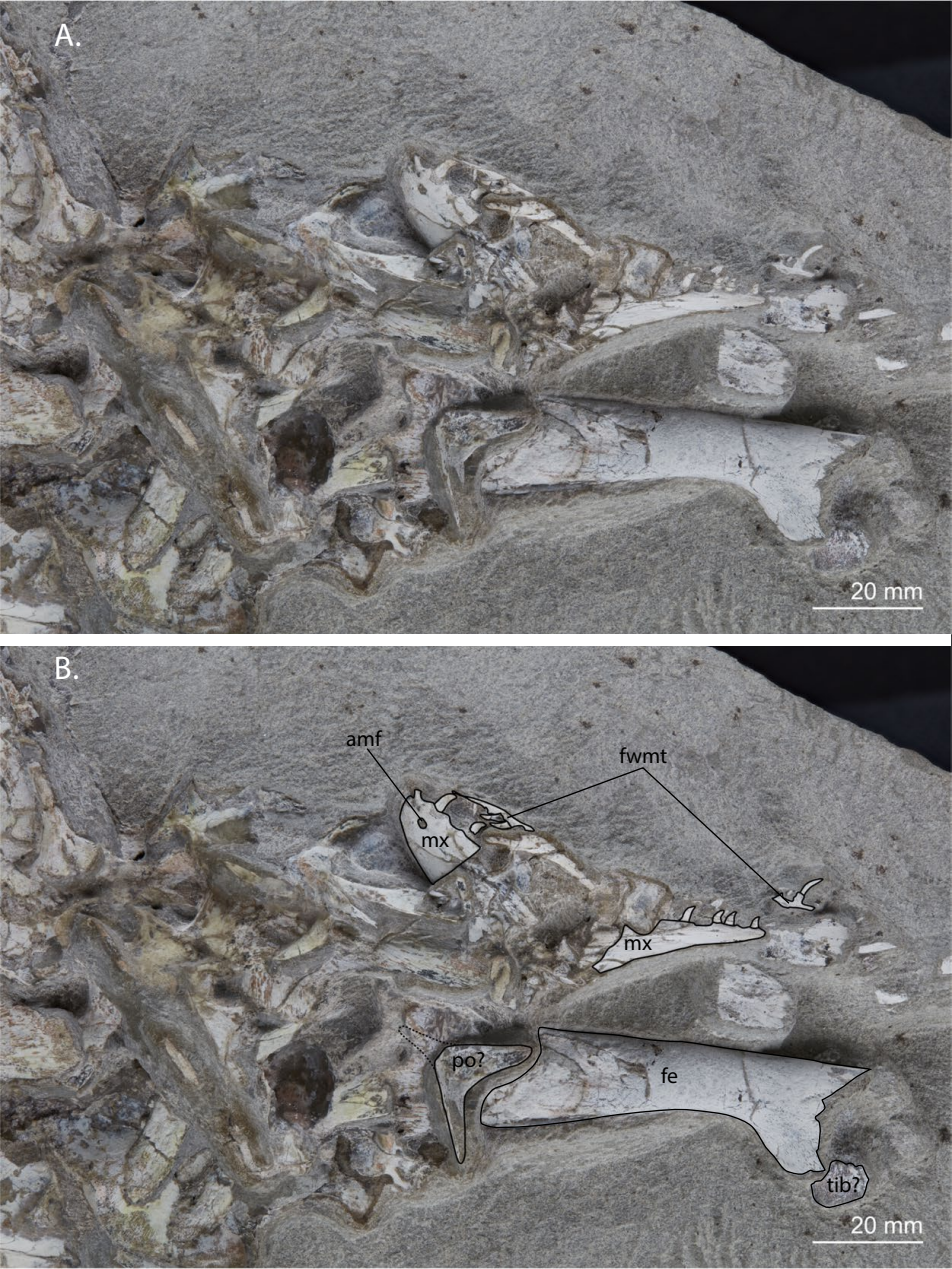
Close-up view of the region preserving the maxilla, postorbital(?), and several fragments of the skull region of UWBM 95529. (**A**) Photograph and (**B**) photograph including line drawings. Abbreviations used in the figure are anterior maxillary foramen (amf), femur (fe), fragments with marginal teeth (fwmt), maxilla (mx), postorbital (po), and tibia (tib).

**Figure 3:**
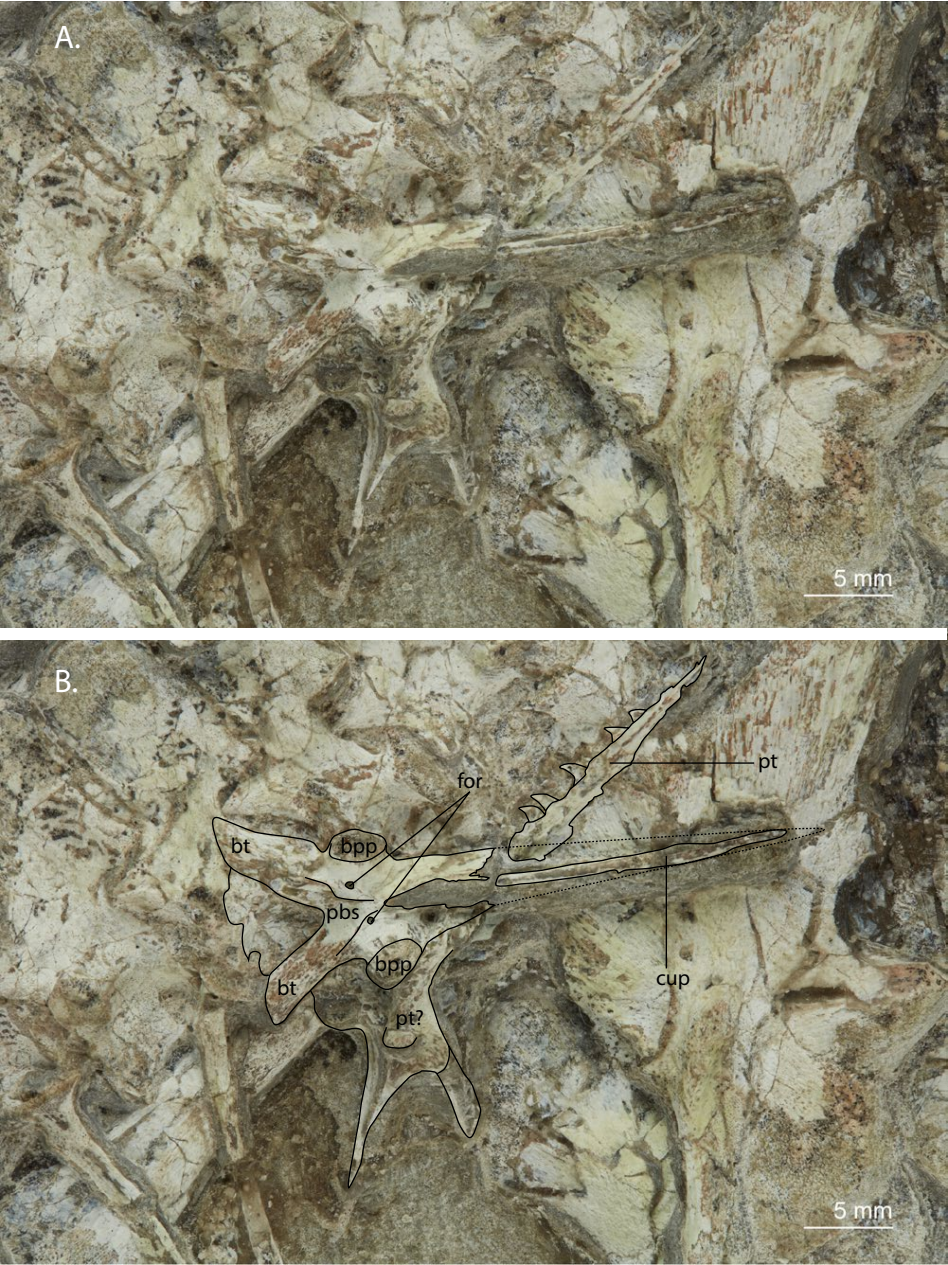
Close-up view of the parabasisphenoid and pterygoid of UWBM 95529. (**A**) Photograph and (**B**) photograph including line drawings. Abbreviations used in the figure are basipterygoid process (bpp), basal tuber (bt), cultriform process (cup), foramen (for), parabasisphenoid (pbs), and pterygoid (pt).

**Figure 4:**
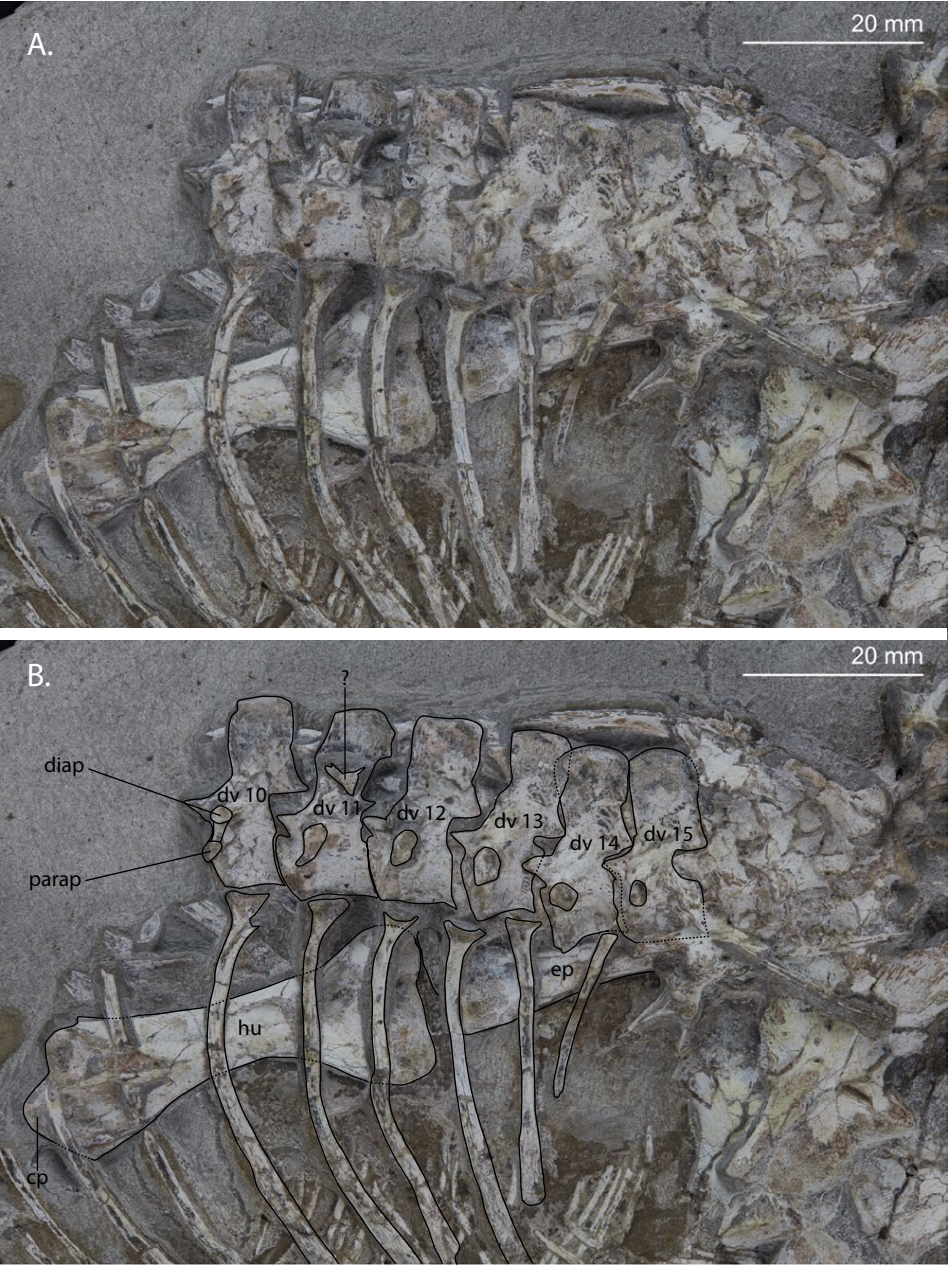
Close-up view of the dorsal vertebral column and left humerus of UWBM 95529. (**A**) Photograph and (**B**) photograph including line drawings. Abbreviations used in the figure are conical process of the humerus (cp), diapophysis (diap), dorsal vertebra (dv), epipodial (ep), humerus (hu), and parapophysis (parap).

**Figure 5:**
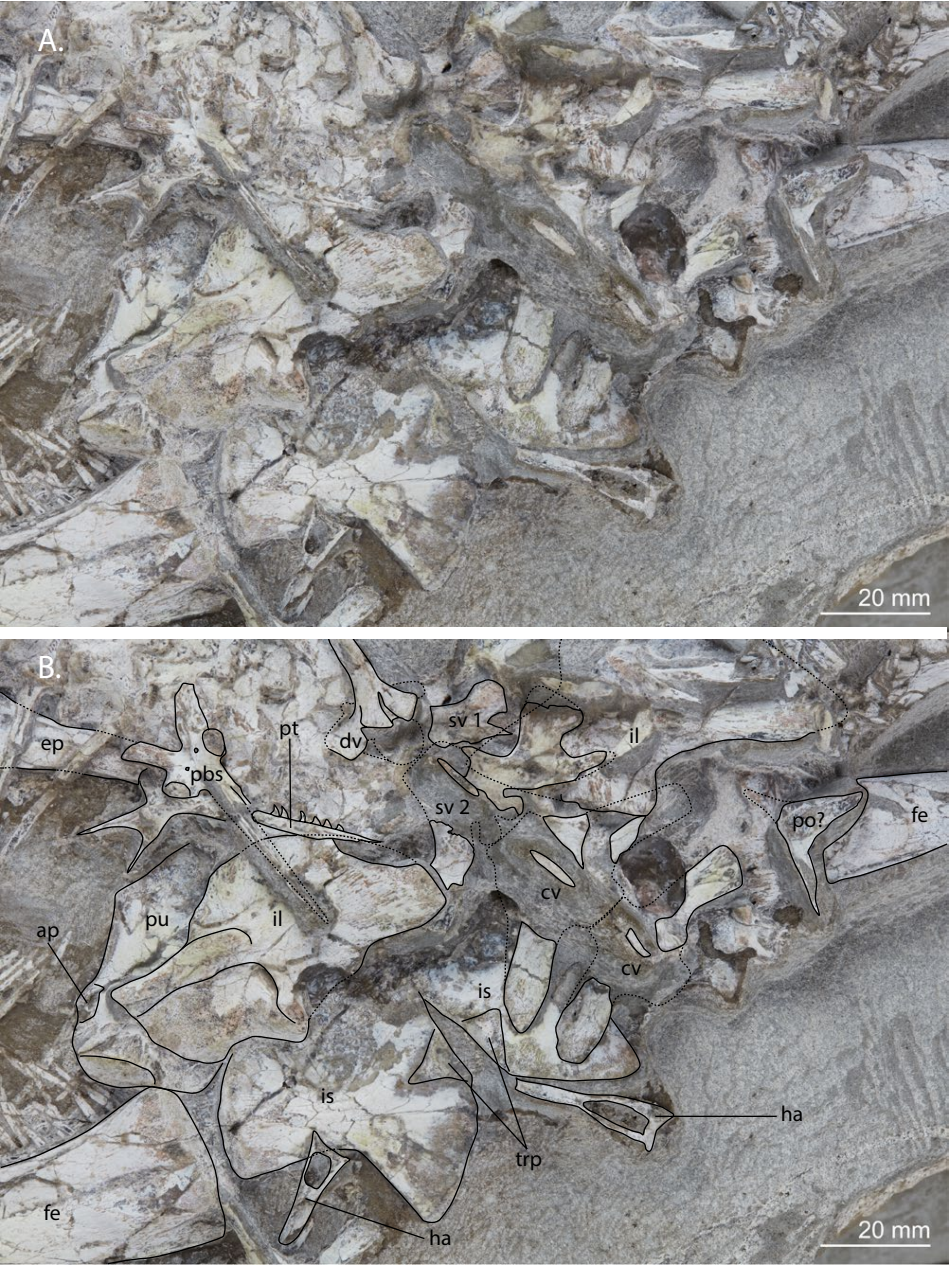
Close-up view of the pelvic region of UWBM 95529. (**A**) Photograph and (**B**) photograph including line drawings. Abbreviations used in the figure are ambiens process (ap), caudal vertebra (cv), dorsal vertebra (dv), epipodial (ep), femur (fe), haemal arch (ha), ilium (il), ischium (is), parabasisphenoid (pbs), postorbital (po), pterygoid (pt), pubis (pu), sacral vertebra (sv), and triangular peduncle (trp).

The presence of septomaxillae is generally hard to determine since it is a small bone that is easily lost during preservation and a broken off piece of maxilla or other bone can easily be mistaken for it. Nevertheless an element which very likely is a septomaxilla can be observed in UCMP 37151 (University of California Museum of Paleontology, USA)^9^. Among other archosauromorphs, septomaxillae have only been reported for *Trilophosaurus buettneri* and *Proterosuchus* (Ezcurra, character 45)^10^, and very tentatively for *Tanystropheus longobardicus*^26^. The presence of recurved maxillary teeth is a character that *Prolacerta* shares with *Macrocnemus*, *Tanystropheus longobardicus*, and *Boreopricea funerea* among non-archosauriform archosauromorphs (Fig. 2; Ezcurra, character 303)^10^. The presence of a long horizontal dorsal margin of the maxilla with a posteriorly concave margin is a character that is only observable in *Prolacerta broomi* (e.g. BP/1/5375, Evolutionary Studies Institute, formerly Bernard Price Institute for Palaeontological Research, University of Witwatersrand, South Africa) and *Teyujagua paradoxa*^14^. In contrast, in archosauriforms, the presence of an antorbital fenestra results in a much narrower dorsal (=ascending) process of the maxilla, whereas in other non-archosauriform archosauromorphs the dorsal margin of the maxilla is either orientated anteroventrally to posterodorsally with a concave posterior margin (tanystropheids^21,27^, allokotosaurs^28^, and rhynchosaurs^29,30^), or a concave posterior margin is absent (e.g. *Protorosaurus speneri*)^31^. Posterolateral processes on the frontals can be observed in various *Prolacerta* specimens (e.g. BP/1/471). Similar processes can also be found in *Macrocnemus*, *Jesairosaurus lehmani*, and *Kadimakara australiensis* among non-archosauriform archosauromorphs (Ezcurra, character 116)^10^. The combined presence of foramina and crests on the ventral body of the parasphenoid, which is observed in *Prolacerta* (Fig. 3), is also found in *Proterosuchus fergusi* and *Mesosuchus browni* (*Proterosuchus*: BP/1/3993, *Mesosuchus*: SAM-PK-6536, Iziko South African Museum, South Africa; Pritchard *et al*., characters 66 and 67)^13^. The presence of medially directed teeth on the medial margin of the pterygoid observed in *Prolacerta* (Fig. 3), has also been reported for *Boreopricea funerea*, *Proterosuchus*, and *Tasmaniosaurus triassicus* (Ezcurra, character 199)^10^. The conical process on the proximal end of the humerus present in *Prolacerta* (Fig. 4) can also be observed in *Trilophosaurus buettneri*, *Pamelaria dolichotrachela*, and *Shringasaurus indicus* (Ezcurra and Sengupta *et al*., character 420)^10,32^. A tuberosity on the anterior margin of the pubis that represents the ambiens process can be observed in UWMB 95529 (Fig. 5). A similar tuberosity among non-archosauriform archosauromorphs was also reported for *Azendohsaurus madagaskarensis*, *Pamelaria dolichotrachela*, and *Shringasaurus indicus* (Ezcurra and Sengupta *et al*., character 474)^10,32^. A triangular peduncle on the medial side of the ischium can also be observed in UWBM 95529 (Fig. 5). This character was previously thought to be unique to certain allokotosaurs and known to be present in *Azendohsaurus madagaskarensis* and *Pamelaria dolichotrachela* (Ezcurra and Sengupta *et al*., character 486)^10,32^.

### Age and paleogeographic range

Earliest Triassic (Induan) of South Africa and Antarctica^4,33^.

### Holotype

**UMZC 2003.40** (University Museum of Zoology, Cambridge, UK), a partial skull and mandibles.

### Hypodigm

South African specimens referred to *Prolacerta broomi*: **UCMP 37151**, complete skull and mandibles and cervical vertebrae 1–6, **BP/1/471**, complete skull and mandibles and anteriormost cervical vertebrae; **BP/1/2675**, acid prepared, disarticulated, partial skull and anterior postcranial skeleton including forelimbs; **BP/1/2676**, articulated vertebral column ranging from the anterior cervical vertebrae to the anterior tail region, as well as multiple disarticulated limb and girdle elements; **BP/1/4504a**, complete skull and mandibles and anterior part of the neck; **BP/1/5066**, partial skull missing the snout; **BP/1/5375**, partial skull and mandibles missing the snout; **BP/1/5880**, complete skull and mandibles and anterior part of the neck; **NMQR 3753** (National Museum, Bloemfontein, South Africa), skull and anterior skeleton in nodule; **NMQR 3763**, two skulls and a curled up skeleton; **SAM-PK-K10620**, two partially articulated skeletons preserved together with a *Dicynodon* humerus; **SAM-PK-K10797**, skull and mandible; **SAM-PK-K10801**, a few disarticulated bones; **SAM-PK-6539**, proximal part of a humerus; **SAM-PK-6541**, articulated pelvic region including sacral vertebrae; **SAM-PK-K10439**, partial skull; **SAM-PK-K10018**, complete skull and mandibles and some indeterminable small fragments; **SAM-PK-K10797**, complete skull and partial mandibles, as well as a partial long bone; **SAM-PK-K10802**, nodule containing a partial skull, mandibles, and skeleton; **SAM-PK-K10965**, various fragmented elements, including small parts of the skull and mandibles; **SAM-PK-K10978**, partial skull and mandibles articulated with the anterior cervical region, as well as some isolated elements; **SAM-PK-K11523**, skull preserved in smooth grey nodule with a piece of the skull roof missing; **SAM-PK-K11520**, recently prepared with no further information available yet; **RS659 (field number)**, newly found specimen not yet accessioned.

Antarctic specimens referred to *Prolacerta broomi*: **UWBM 95529**, skeleton with a few skull remains (maxilla, postorbital, parabasisphenoid, and pterygoid) and most of the postcranium intact, only missing the cervical and anterior dorsal part of the vertebral column, the left forelimb, the distal part of the right hind limb, and the posteriormost part of the tail; **AMNH 9520**, fully preserved skull in left lateral view; **AMNH 9521**, fully preserved, partially disarticulated skull in ventral view; **AMNH 9502**, partially articulated specimen including a premaxilla, some fragmentary skull bones and teeth, a partial vertebral column including a poorly preserved sacral region and large part of the tail, a poorly preserved pelvic region, and two hind limbs, of which the right one is well preserved, including an articulated foot; **AMNH 9551**, partial humerus with the distal end preserved and some bone fragments; **AMNH 9557(?*Prolacerta*)**, a few bone fragments and a damaged humerus. The distal end of the humerus is less wide than in AMNH 9551, but this could be due to the angle of preservation, since the bone is flattened; **AMNH 9558**, skull fragments including parts of the mandibles, maxillae, premaxillae, and a palatine, as well as two cervical series, indicating two different individuals are represented in the specimen(?) and some isolated metacarpals or phalanges; **AMNH 9560**, partially preserved hindlimb, as well as a poorly preserved vertebral series, and some bone fragments, including skull fragments with marginal teeth; **AMNH 9564**, partial cervical and dorsal series, including some dorsal ribs and long bone fragments; **AMNH 9568**, well preserved pterygoid, ectopterygoid, and cervical vertebra, as well as some bone fragments; **AMNH 9573(?*Prolacerta*)**, partial forelimb including pectoral girdle and a number of bone fragments; **AMNH 9574**, three identifiable dorsal vertebrae and a number of bone fragments, including long bones; **AMNH 9526**, number of very fragmented bones, including a bone preserving marginal dentition, most likely a maxilla; **AMNH 9561**, specimen currently not present at the AMNH and was therefore not evaluated personally by the author. Colbert described the specimen as follows: “Ten articulated presacral vertebrae, with ribs; also caudal vertebra. Various other bones, including paired ischia (seen ventrally), part of a femoral shaft, a fragment of a scapulacoracoid with the proximal end of crushed humerus, and a jaw fragment with four alveoli”^1^. **AMNH 9519**, a specimen not discussed by Colbert^1^ although it was collected during the same field trips as the other specimens he described. It was found in 1970–1971 field season at Kitching Ridge, Fremouw Formation, Antarctica by James W. Kitching. The remains are poorly preserved and likely represent at least two different specimens. Some gastralia, a partial manus or pes, tail, and presacral vertebral series are identifiable. The wide phalanges of the manus or pes coincide with the morphology seen for *Procolophon trigoniceps*^34^. However the tail deviates from the morphology of *Procolophon*, and is indistinguishable from the tail morphology known for *Prolacerta* with comparitively long and slender haemal arches. The presacral vertebrae preserve a morphology that fits both the known morphology of the cervical vertebrae of *Procolophon* and the dorsal vertebral morphology of *Prolacerta*.

### Remarks

A large number of the specimens listed above are currently undescribed. However, many of them were mentioned in the Supplementary Table of Smith & Botha-Brink^35^. Furthermore, a number of additional specimens were also referred to *Prolacerta* by Colbert^1^ but are here considered to be too fragmentary to be confidently identified as *Prolacerta*. These are specimens: **AMNH 9522**, a bundle of very fragmented cranial and postcranial bones, of which one bone preserves the marginal dentition. The teeth on this bone are very closely packed which is not the case in *Prolacerta*, indicating this specimen might belong to a different tetrapod taxon; **AMNH 9552**, a number of very poorly preserved, fragmented bones that do not allow for a confident identification; **AMNH 9513**, a partial maxilla with teeth, of which both the morphology of the bone as well as the teeth do not fit *Prolacerta*. An identification will not be provided here, but the label suggests it might be a millerettid; **AMNH 9535**, six, likely dorsal, vertebrae in articulation. However, the preservation of the vertebrae is so poor that it does not allow for the observation of any potentially diagnostic characters.

## Description of UWBM 95529

UWBM 95529 was collected at Graphite Peak, locality C1589, the lower Fremouw Formation (Induan, Early Triassic)^33^, Antarctica. It comprises an articulated skeleton, including four identifiable skull bones, a vertebral column missing the cervical and anterior dorsal vertebrae and parts of the tail, a nearly complete right forelimb including parts of the pectoral girdle, the humerus of the left forelimb, a left hind limb including the left pelvic girdle missing some tarsal elements and the phalanges, and a right femur and parts of the right pelvic girdle (Fig. 1).

### Skull

A number of skull bones have been preserved, of which the maxilla, postorbital, parabasisphenoid, and pterygoid can be identified.

#### Maxilla

A right maxilla is preserved in lateral view and located next to the right femur (Fig. 2). It is broken in two pieces which are no longer aligned. The surface of the anterior part is slightly convex and clearly bears an anteriorly opening foramen, the anterior maxillary foramen. The posterior part of the maxilla tapers posteriorly and bears teeth all the way to its end. Its dorsal margin is straight. The maxillary teeth are typical of *Prolacerta*, being recurved and slighty labiolingually compressed without any serrations or striations. However, in contrast to other *Prolacerta* specimens, both from Antarctica (AMNH 9520 and AMNH 9521) and South Africa (e.g. BP/1/471, UCMP 37151, and SAM-PK-K10018), the teeth of UWBM 95529 are much smaller in comparison to the overall body size. Two teeth are present on the anterior part of the maxilla and four on the posterior part, as well as three teeth on two isolated fragments that are parts of the premaxilla, maxilla, or dentary.

#### Postorbital(?)

Remains of skull roof bones are preserved, but their fragmentary nature makes confident identification impossible. However one bone could be tentatively identified as a postorbital, of which the posterior process is broken off (Fig. 2). It is crescent-shaped and one end, the dorsal end, is distinctly narrower than the ventral end. The surface of the bone is somewhat concave, which would indicate that the bone is preserved in medial view, in which case it is the right postorbital.

#### Parabasisphenoid

A parabasisphenoid is preserved in ventral view and partially overlying the left ilium (Fig. 3). Its cultriform process is long and thin and is ventrally broken across almost its entire length. Although it cannot be determined unequivocally because of this, the cultriform process does not appear to be as dorsoventrally compressed as in the South African specimens BP/1/2675 and BP/1/5066. The basipterygoid processes point somewhat anterolaterally but are mainly laterally orientated. The basal tubera are clearly separated. Two foramina for the passage of the internal carotid arteries are clearly present on the parabasisphenoid body adjacent to the basipterygoid processes. Directly medial to each foramen a crest runs posterolaterally, forming the margins of the floor of the vidian canal.

#### Pterygoid

Directly below and next to the parabasisphenoid a partial pterygoid is preserved (Fig. 3). It is a long and thin bone with a row of teeth on its medial side. The teeth are recurved and considerably smaller than the teeth of the maxilla. The bone is broken laterally and is posteriorly covered by the parabasisphenoid. On the other side of the parabasisphenoid a partially broken element is preserved which might represent the posterior end of the pterygoid. In this case the entire bone would likely be the left pterygoid in ventral view, being still in close association with the basipterygoid process of the parabasisphenoid. It bears two pointed processes, which might be a remnant of the lateral ramus and a part of the broken off posterior ramus. However, two bulbous structures are present on the main body of the element, which cannot be identified and do not occur on the pterygoids of any other *Prolacerta* specimen in which this element is visible (BP/1/5066, AMNH 9568, and UCMP 37151).

### Axial skeleton

No cervical or anterior dorsal vertebrae are preserved. However, the vertebral column, including ribs and gastralia, is intact and in articulation from the 10th dorsal up to the 2^nd^ caudal. Furthermore, the distal part of the tail is also in articulation and a number of isolated proximal and distal caudals are present. The dorsal vertebrae are visible in left lateral view. However the three most posterior dorsals are visible in dorsolateral view with each being orientated more dorsally than the preceding one. The sacrals and proximal caudals are evident in dorsal view, and one of the caudals is seen in posterior view. No intercentra are present. A large number of gastralia are preserved, as well as a partial vertebral column of another animal that is likely stomach content of the *Prolacerta* specimen.

#### Dorsal vertebrae and ribs

The dorsal ribs of the left side have been disarticulated but are in close association with their respective vertebrae (Fig. 4). Two elongate bones are present behind the neural spines of dorsals 10 to 12 and one above dorsals 13 to 15. The former two might be right dorsal ribs, however the latter seems to have a different morphology, being very flat and wide. Its upward projecting margin is straight but clearly not complete and its lower margin is curved downward. Directly adjacent to it, another bone fragment is present which might be part of the broken off neural spine of dorsal 16, on top of which it is lying. Any other dorsal ribs of the right side are not visible. The parapophysis and diapophysis of dorsal 10 can clearly be distinguished and are connected by a bony lamina. Because the proximal part of the associated rib is poorly preserved, it is unclear whether the rib had a distinct capitulum and tuberculum or one confluent head. In the subsequent two dorsals the parapophysis shifts more dorsally towards the diapophysis until they become fully confluent in dorsal 13. The rib of dorsal 14 is somewhat shorter than the preceding ribs. The rib of dorsal 15 is much shorter than the rib of dorsal 14, indicating a distinct lumbar region in *Prolacerta*. No ribs can be observed for dorsals 16 to 18, but these could be covered by the overlying bones of the skull and the pelvic girdle. The neural spines of the dorsals are slightly higher than they are anteroposteriorly long and are slightly longer distally than at their base. A small fossa can be observed at the base of the neural spine. The nature of the articulation of the centra cannot be established. A small unidentifiable bone fragment is preserved on top of the neural spine of dorsal 11.

#### Gastralia and stomach contents

**Figure 6:**
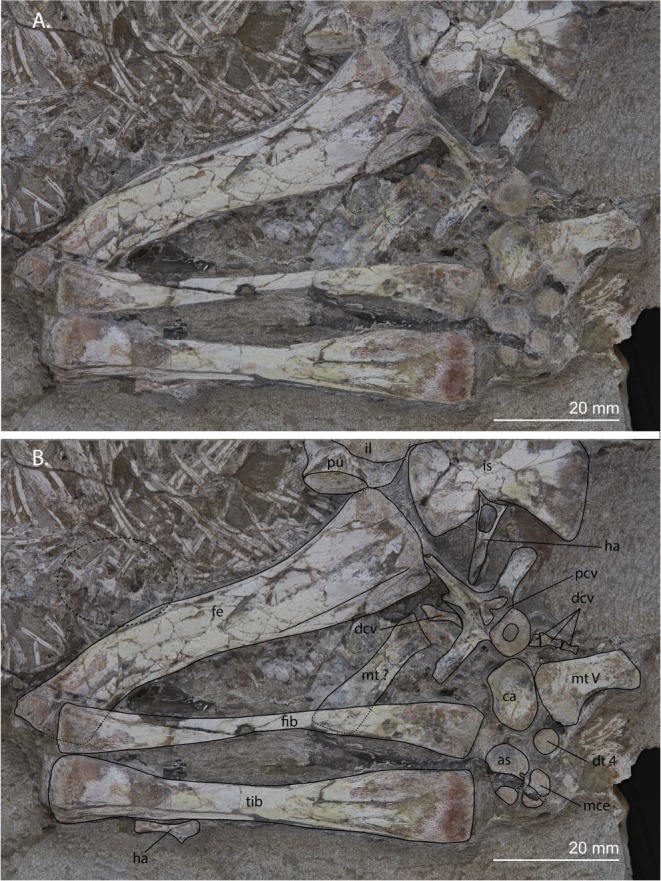
Close-up view of the left hind limb as well as the partial vertebral column that constitutes stomach contents of UWBM 95529. (**A**) Photograph and (**B**) photograph including line drawings. The dashed ellipse encloses the four vertebrae that are part of the stomach contents. Abbreviations used in the figure are astragalus (as), calcaneum (ca), distal caudal vertebra (dcv), distal tarsal (dt), femur (fe), fibula (fib), haemal arch (ha), ilium (il), ischium (is), medial centrale (mce), metatarsal (mt), proximal caudal vertebra (pcv), pubis (pu), and tibia (tib).

A large number of gastralia are preserved that are closely packed and pointing anteromedially. Additionally, below the dorsal ribs and among the gastralia, close to the left hindlimb, a partial vertebral column can be observed (Fig. 6). It comprises eroded centra, as well as probable neural arches, and transverse processes/ribs that are fused to the vertebrae. The presence of these processes excludes the possibility that these are distal caudals of the individual and they are too small to belong to any other of its vertebrae. Therefore they must represent another individual vertebrate, likely a tetrapod. However, not enough detail is preserved to make a more precise identification. Their position among the gastralia makes it highly likely they represent stomach contents.

#### Sacral vertebrae

Two sacral vertebrae with their transverse processes/ribs are present, of which the second is the best preserved (Fig. 5). The transverse process/rib of the second sacral is somewhat longer than the transverse process/rib of the first sacral and bifurcated.

#### Caudal vertebrae

**Figure 7:**
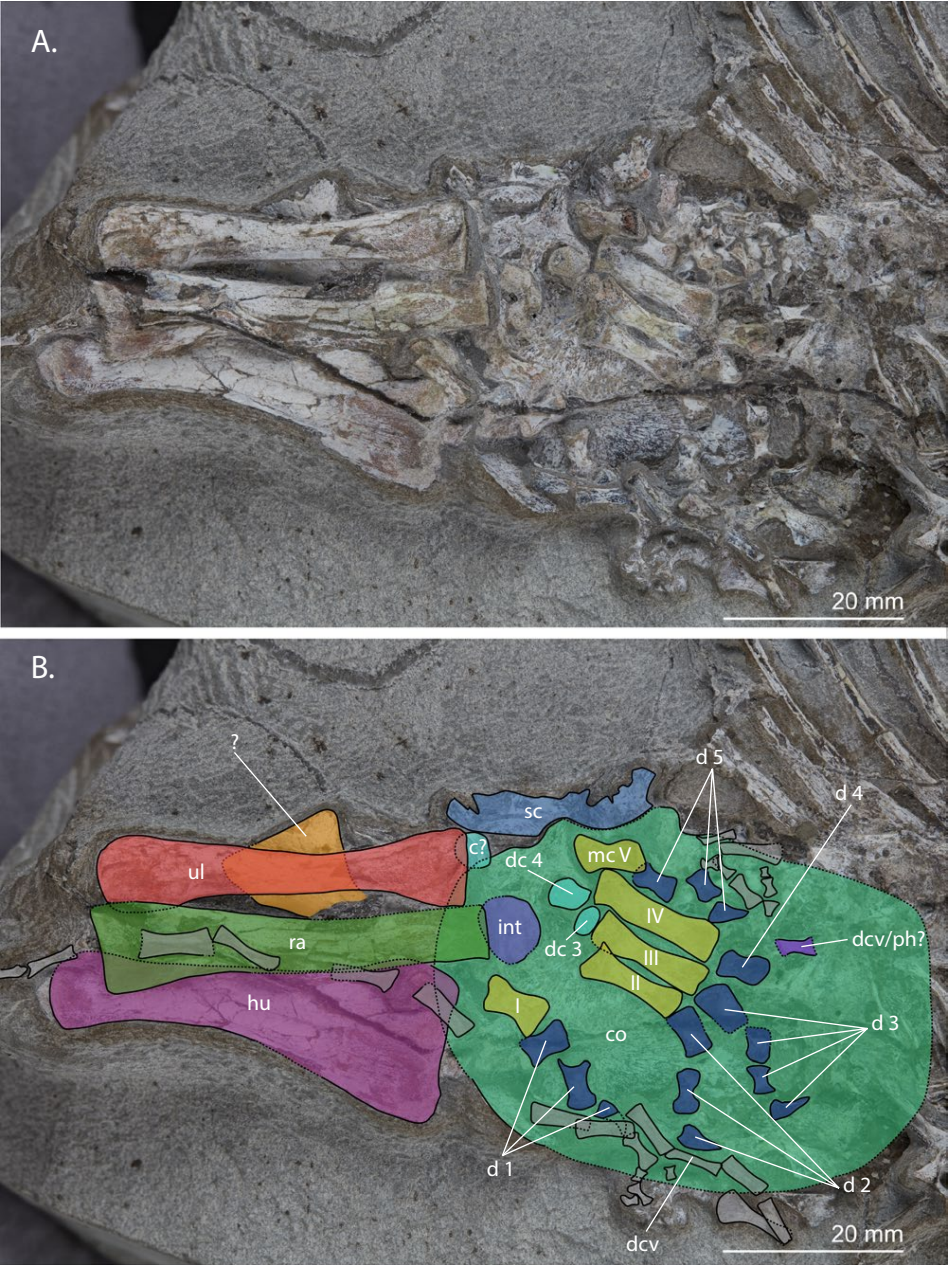
Close-up view of the right forelimb and partial pectoral girdle of UWBM 95529. (**A**) Photograph and (**B**) photograph including line drawings. Abbreviations used in the figure are carpal (c), coracoid (co), digit (d), distal carpal (dc), distal caudal vertebra (dcv), humerus (hu), metacarpal (mc), phalanx (ph), radius (ra), scapula (sc), ulna (ul), and intermedium (int).

Articulated with these two sacrals are two anterior caudals (Fig. 5). Their transverse processes are orientated somewhat posterolaterally. Two other anterior caudals are present but disarticulated; one is located between the left ischium and pes and the other close to the gastralia. The former is preserved in posterodorsal view, the latter in posterior view. The posterior surfaces of their centra are concave and lack an opening for the notochordal canal. Their neural spines are tall, being distinctly taller than anteroposteriorly long. Three isolated haemal arches of anterior caudal vertebrae are also preserved (Figs 5 and 6). Twelve distal caudals are preserved in articulation and are lying on top of the right forelimb. At least eight more disarticulated distal caudals are preserved next to manual digit V (Fig. 7).

#### Appendicular skeleton. Pectoral girdle

The coracoid and a fragment of the scapula(?) of the right forelimb are preserved below the long bones and manus of the same limb (Fig. 7). A clear suture is visible between the partial scapula and the coracoid. The coracoid is plate-like and most likely preserved in medial view, since no glenoid fossa can be discerned. Directly posterior to the scapulacoracoid suture is a small dorsally directed process on the coracoid, which is likely homologous to the process posterior to the scapulacoracoid suture in BP/1/2675. Ventral to the scapulacoracoid suture a coracoid foramen is present. The overall shape of the coracoid cannot be discerned because of the overlying bones.

### Humerus

Both humeri are completely preserved with their deltopectoral crests facing upward from the slab and are therefore in anterior view. The right humerus is partially covered by the radius and distal caudal vertebrae with the proximal end in close association with the coracoid (Fig. 7). The left humerus is located underneath the dorsal ribs but presents more detail than the right humerus (Fig. 4). There is clearly some torsion in both humeri but much less than observed in the humerus of BP/1/2675. A conical process can clearly be observed on the proximal end of the left humerus. No foramina are present on the distal end of either of the humeri. The entepicondyle is similar in size to the ectepicondyle and no supinator process and groove can be observed.

### Radius and ulna

The proximal end of one epipodial of the left forelimb is present (Fig. 4). It is not identifiable based on its morphology, but its close association with the entepicondyle of the humerus might suggest it is the left ulna. Both the right radius and ulna are completely preserved (Fig. 7). They are subequal in length, the radius being slightly longer than the ulna (Table 1). The ulna lacks an olecranon process. A flat, plate-like bone, which cannot be identified, is preserved below the radius and ulna. The orientation in which both bones are preserved cannot be deduced based on their morphology alone. However, the close association with the metacarpals indicates that they are likely preserved in the same orientation and are thus probably exposed in posterior view.

**Table 1:** Measurements taken from the limb bones of UWBM 95529.

Right Humerus	48.1 mm
Left Humerus	48.9 mm
Right Radius	44.9 mm
Right Ulna	43.0 mm
Metacarpal I	7.3 mm
Metacarpal II	12.6 mm
Metacarpal III	13.9 mm
Metacarpal IV	15.7 mm
Metacarpal V	7.5 mm
Left Femur	72.4 mm
Left Tibia	69.3 mm
Left Fibula	68.9 mm
Metatarsal V	17.6 mm

#### Manus

Largely on top of the right coracoid and at the proximal end of the right humerus and distal ends of the right radius and ulna, a largely articulated right manus is preserved (Fig. 7). Four carpal bones are present. A large, partially broken carpal is located at the distal end of the radius and likely represents the intermedium. Another carpal element is preserved partially below the distal end of the ulna. Although its position directly distal to the ulna could potentially indicate it to be an ulnare, its smaller size compared to the intermedium disputes this notion and suggests that it represents either a shifted centrale or a distal carpal. The other two carpal bones, which are roughly similar in size to the previously mentioned bone, are located close to metacarpals III and IV and are therefore identified as distal carpal three and four. Metacarpals II-V are preserved in their natural articulation in ventral view. Metacarpal I has shifted somewhat away from the other metacarpals. Metacarpal I and V are much shorter than the other three metacarpals, of which metacarpal IV is the longest (Table 1). Digit I is somewhat disarticulated but complete, with two complete phalanges distal to metacarpal I and a broken phalanx which almost certainly is an ungual distal to the preceding phalanges. Three phalanges can be observed distal to metacarpal II, of which the distalmost is an ungual. Phalanx I of digit III is distally broken. Distal to it another three phalanges are preserved, of which the distalmost is an ungual. Phalanx I of digit IV is intact. A broken phalanx II might be located distally to this phalanx, but its identification is equivocal and it might also be a distal caudal vertebra. No other phalanges of digit IV are observable. Digit V appears to be complete and in articulation, with two non-ungual phalanges and one ungual. All unguals are of narrow triangular shape and more or less similar in size to their respective preceding phalanges.

#### Pelvic girdle

Of the right side of the pelvic girdle the ilium and ischium can be identified (Fig. 5). The ilium is preserved in medial view and covered by fragments of ribs and skull bones. Its postacetabular process extends towards the anterior part of the maxilla. The ischiac blade is visible underneath and next to the left transverse processes of the two anteriormost caudals. The rest of the ischium is covered beneath the sacral and anterior caudal vertebrae. It is also preserved in medial/dorsal view. The left side of the pelvic girdle is fully preserved. The ilium is visible in lateral view and covers most of the pubis and part of the ischium, which are both preserved in medial/dorsal view. The surface of these pelvic bones has been damaged, allowing the observation of dorsoventral striations, particularly on the postacetabular process of the ilium, which are osteological correlates of arteries and nerves. It is unclear whether the posterior part of the postacetabular process is complete or partially broken off. The anterior margin of the ilium is convex and does not have a small spur-like process as seen in the ilia of certain tanystropheids (e.g. *Macrocnemus bassanii* PIMUZ T 2472, Paleontological Institute and Museum of the University of Zurich, Switzerland, and *Tanystropheus longobardicus* PIMUZ T 1277), but a small concavity is present on the anterior margin dorsal to the supra-acetabular crest. The acetabulum is largely located on the ilium, which bears a prominent supra-acetabular crest. The iliac margin directly posterior to the acetabulum has broken off. Although it is partially covered by the cultriform process of the parabasisphenoid, a brevis shelf can clearly be made out on the ilium. Most of the pubis is covered by the ilium and only the anterior margin is visible, which bears a tuberous protrusion for the attachment of the ambiens muscle. On the distal end of the ischium, a small triangular peduncle is present, which formed part of the symphysis with the other ischium. The posterior margin of the ischium is linear and makes an angle of approximately 90 degrees with the ventral margin, which is convex. The anterior margin is not visible and although it could be present below the ilium, it is likely fused to the pubis, meaning no thyroid fenestra was present.

### Femur

The right femur is preserved and its proximal end is largely obscured by the overlying postorbital(?) and the right transverse process of caudal vertebra 2 (Figs 2 and 5). It is preserved in right lateral to ventral view. Because the surface of the proximal half of the bone is broken, no fourth trochanter is visible. The distal end is largely broken off and the distal condyles are not preserved. A small fragment of bone is present distal to the femur and based on its position close to the medial condyle of the femur it might represent a part of the tibia. The left femur is complete and preserved in left lateral view (Fig. 6). A proximodistal distal groove runs through the middle of the shaft, indicating the bone has been crushed during preservation. It is sigmoidal in shape in lateral view. The fourth trochanter is clearly present and runs parallel to the shaft. The distal end is partially covered by the fibula but the condyles do not seem to expand much from the shaft.

### Tibia and fibula

The left tibia and fibula are both fully intact, slender, and symmetrical (Fig. 6). The fibula is distinctly narrower than the tibia. Both bones are almost identical in length and slightly shorter than the femur (Table 1). Although the preserved morphology does not allow for identifying the orientation of the bones, similarly as for the radius and ulna, their orientation can be deduced based on their articulation with the pes. Since the pes is preserved in dorsal view, it is likely both bones are seen in anterior view.

#### Pes

The right pes is not preserved. The left pes, of which four tarsal bones are preserved, is seen in dorsal view (Fig. 6). The best preserved tarsal element is the calcaneum, which is the large, circular bone between the fibula and metatarsal V. Its proximal and mediodistal margins are somewhat raised. The margin of the perforating foramen is likely present but cannot be seen on the calcaneum. Medial to the calcaneum is the astragalus. It is distinctly wider than it is proximodistally long. It is broken through the middle as the specimen was split in two during excavation and later glued together (pers. comm. Brandon Peecook and Christian Sidor). The margin of the perforating foramen is preserved on the astragalus, where it is formed on its distal end by a small concavity. The larger of the two other bones is located between the distal margins of the astragalus and calcaneum and is therefore almost certainly distal tarsal 4; its proximal end points upward somewhat. The other bone is located laterodistally to the astragalus and is also broken in half by the same crack that split the astragalus. It is rounded and likely is the medial centrale. Metatarsal V is the only metatarsal that is still in its natural articulation. It is somewhat hook-shaped, but the angle of the medial margin between the proximomedial process and the rest of the metatarsal is quite low. The lateral margin of metatarsal V is very straight and smooth. Another bone, located between the distal end of the fibula and the proximal end of the femur, is probably a metatarsal, but because it has shifted from its original position and is poorly preserved it is impossible to identify. Its length cannot be measured since it is partially covered by the fibula.

## Discussion

### Comparison with other genera

Currently only four reptilian taxa are known from the lower Fremouw Formation which, besides *Prolacerta*, are *Procolophon trigoniceps* and *Palacrodon broomi*, as well as a large unidentified archosauromorph^5,36–38^. Based on the presence of pointed, recurved marginal teeth in the maxilla, UWBM 95529 can be separated from the enigmatic *Palacrodon* and the parareptilian *Procolophon*, which have transversely wided acrodont maxillary teeth^39^ and conical to transversely widened maxillary teeth^40,41^, respectively. Its size, although being quite large for a specimen of *Prolacerta*, is considerably smaller than that of the unidentified archosauromorph, of which only a partial humerus and single vertebra are known. In addition, UWBM 95529 bears a number of characters known for *Prolacerta broomi*, such as the presence of medially directed teeth on the medial margin of the pterygoid (e.g. BP/1/5066 and AMNH 9568), the presence of two foramina and crests on the ventral surface of the parasphenoid (e.g. BP/1/5066), and the presence of a conical process on the proximal end of the humerus (e.g. BP/1/2675). However, since the lower Fremouw Formation is not as well sampled as the very similar AZs of the Karroo of South Africa, from which a much larger diversity of reptiles is known, the possibility of the specimen belonging to a taxon hitherto unknown from the lower Fremouw formation cannot be excluded. The absence of most cranial bones prevents an easy identification of UWBM 95529, since most diagnostic characters described for *Prolacerta* and closely related taxa relate to the skull. However the teeth on the medial edge of the pterygoid, which are directed medially rather than ventrally (Fig. 3), is a character trait only found in *Prolacerta*, *Boreopricea funerea*, *Proterosuchus fergusi*, *Proterosuchus goweri*, *Proterosuchus alexanderi*, and *Tasmaniosaurus triassicus* among early archosauromorphs^10^. A character reported to distinguish *Prolacerta* from the non-archosauriform archosauromorph *Boreopricea* that can be observed in UWBM 95529 is the presence of a perforating foramen between the astragalus and calcaneum, which was reported absent in the latter by Benton and Allen^42^. However, Ezcurra did not score this character nor include it in his emended diagnosis for *Boreopricea*, as he considered this character unobservable in the specimen in its current state of preservation^10^. Another distinguishing character present in UWBM 95529 is the presence of a conical process on the proximal end of the humerus (Fig. 4), which is absent in *Boreopricea* (Benton and Allen, Fig. 11, pag. 940)^10,42^ but present in *Prolacerta* (BP/1/2676; Gow, Fig. 23^2^). The combined presence of these traits in UWBM 95529 shows that UWBM 95529 does not belong to *Boreopricea funerea*.

The easiest character to distinguish *Prolacerta* from the archosauriforms *Proterosuchus* and *Tasmaniosaurus* is the absence of an antorbital fenestra. Unfortunately, the maxilla of UWBM 95529 is insufficiently preserved to determine its absence or presence. However, a number of other characters do indicate UWBM 95529 belongs to *Prolacerta* rather than *Proterosuchus* or *Tasmaniosaurus*. In contrast to *Tasmaniosaurus* UWBM 95529 does not possess dorsal neural spines that are distinctly anteroposteriorly longer at their distal end than at their base, a fossa on the dorsolateral surface of the centra of the dorsal vertebrae (Fig. 4), nor serrated maxillary teeth (Fig. 2)^10,43^. The presence of *Proterosuchus*, like *Prolacerta* known from the *Lystrosaurus* AZ of South Africa is perhaps more likely to be expected than any of the other taxa mentioned above, given the close similarities of these strata to the lower Fremouw Formation. Additionally, the large size and stocky limb bones could also indicate that UWBM 95529 represents a proterosuchid rather than a prolacertid. However, there are a number of clear characters that UWBM 95529 shares with *Prolacerta* to the exclusion of all known species of *Proterosuchus*. The shape of the postorbital, provided its identification is accurate, strongly resembles the morphology seen in *Prolacerta* but not that of *Proterosuchus*, in which its ventral process is much longer and thinner than the dorsal one (Fig. 2)^44^. Additionally, like *Tasmaniosaurus*, the maxillary teeth of *Proterosuchus* are serrated and the dorsolateral surface of the centrum of the dorsal vertebrae bears a fossa, which is not the case in UWBM 95529 (Ezcurra, character 354)^10^. Finally, in contrast to *Proterosuchus*, the dorsal vertebrae of UWBM 95529 lack a hyposphene-hypantrum articulation on the posterior end (Fig. 5) (Ezcurra, character 359)^10^ and the entepicondyle of the humerus is not expanded (Fig. 4) (Ezcurra, character 425)^10^.

**Figure 8:**
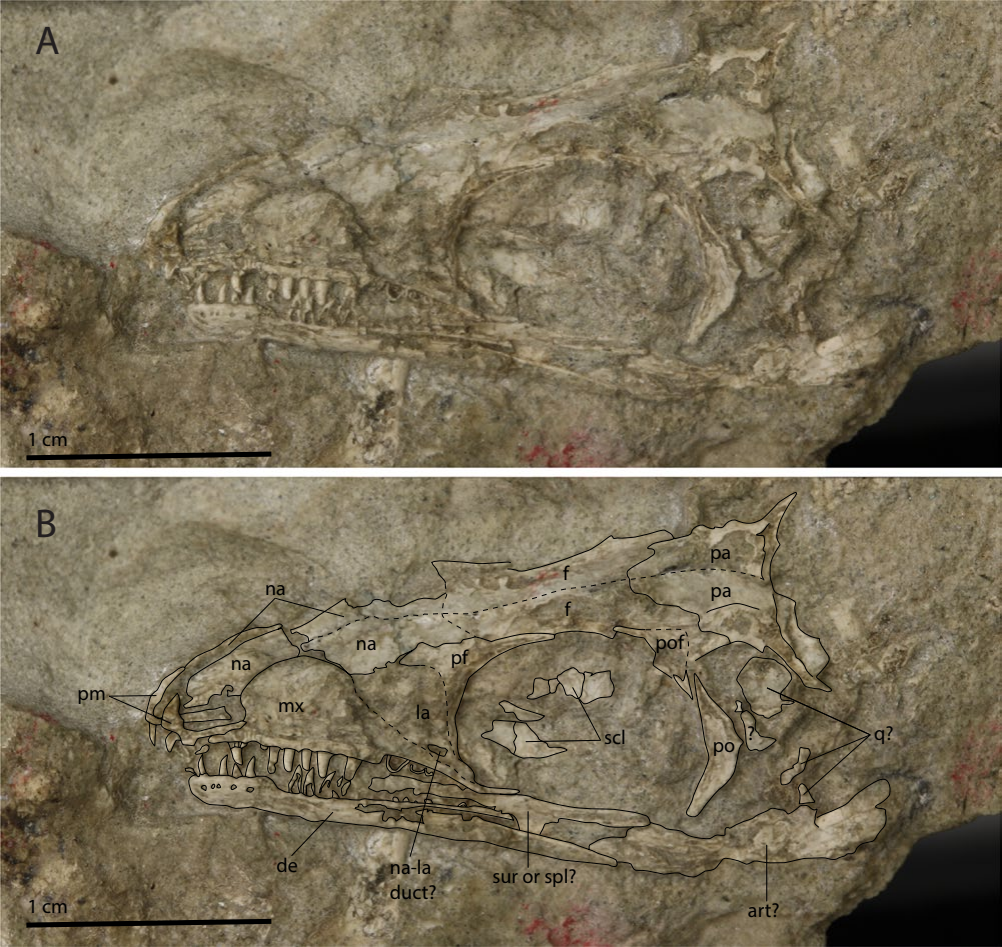
The skull of the juvenile Antarctic *Prolacerta* specimen AMNH 9520. (**A**) Photograph and (**B**) photograph including line drawings. Abbreviations used in the figure are articular (art), dentary (de), frontal (f), lacrimal (la), maxilla (mx), nasal (na), parietal (pa), prefrontal (pf), premaxilla (pm), postorbital (po), postfrontal (pof), quadrate (q), scleral plate (scl), splenial (spl), and surangular (sur).

The combination of these characters in UWBM 95529 leads to the conclusion that its morphology corresponds only to that of *Prolacerta*. However, although the specimen shares no overlapping morphology with this taxon, it is also important to consider *Kadimakara australiensis*^45^. *Kadimakara* is known from the Induan of Queensland, Australia, and two partial skulls have been attributed to the taxon. The morphology of these two specimens is very similar to that of *Prolacerta*, to such a degree that they were considered to be conspecific with *Prolacerta* by some authors^46,47^. However, recent observations indicate that the only difference observable in the currently known material of *Kadimakara* is the presence of a sub-rectangular fossa on the dorsal surface of the posterior half of the parietals^10^. No parietals are preserved in UWBM 95529. However, among the Antarctic prolacertid specimens, a parietal is present in AMNH 9520 (Fig. 8). This parietal does not show a median fossa and has a wide supratemporal fossa, which corresponds to the morphology seen in the South African *Prolacerta* specimens (e.g. BP/1/5375) and contradicts with *Kadimakara*. Therefore the Antarctic specimens are identified as *Prolacerta broomi* rather than *Kadimakara australiensis*.

The assignment of UWBM 95529 to *Prolacerta broomi* implies that it represents the largest known specimen of the species. The more stocky limbs can be explained by the large size of the specimen. However, it is important to note that apart from its large size and consequentially stockier limbs, UWBM 95529 deviates from other *Prolacerta* specimens in two small traits. The marginal dentition is comparatively much smaller than in other known *Prolacerta* specimens and the humerus shows much less torsion than in the only other well-preserved *Prolacerta* humerus known from BP/1/2675. It is possible that because the middle portion of the maxilla is not preserved, which typically bears larger teeth than the anterior and posterior parts of the maxilla (see for instance BP/1/471), UWBM 95529 had larger teeth but that these have not been preserved. The morphology of the humeri, which both show considerably less torsion than the humerus of BP/1/2675, could potentially be the result of compression during preservation, but this seems unlikely. Another more satisfying explanation for this observed discrepancy cannot be provided, but they are considered insufficient to assign the specimen to a new taxon.

### Comparison of the Antarctic material with the South African material of *Prolacerta broomi*

Although Colbert considered the Antarctic material he described to be conspecific with *Prolacerta broomi* from South Africa, he did point out several small morphological differences^1^. He noted that many of these differences are related to proportions that somewhat deviate from those described for *Prolacerta* by Parrington, Camp, and mainly Gow^2,6,9^. These types of characters, particularly when they only show small differences, are problematic for species identification, as they are more likely to represent a form of intraspecific variation due to ontogeny or sexual dimorphism rather than a taxonomic signal^48^. Colbert also pointed out that a number of these differences were likely related to ontogeny^1^. Furthermore, intraspecific variation within the *Prolacerta* material from South Africa was already previously reported, specifically regarding the pineal foramen, which is present in certain specimens, albeit varying in size, and is completely absent in others^2,3^. However, since all specimens he described were distinctly smaller than the specimens from South Africa that were considered adult, it was also plausible that the Antarctic material represented a dwarfed form closely related to *Prolacerta broomi*. Botha-Brink & Smith used skull length as well as midshaft and proximal width of the tibia to establish overall size in *Prolacerta*^19^. Unfortunately the skull of UWBM 95529 is insufficiently preserved to determine its length and because the tibia measurements are small its margin of error is quite large. Instead femur length and femur midshaft width were used to compare the size of UWBM 95529 to NMQR 3763. NMQR 3763 comprises two individuals, of which one was sampled, measured, and discussed by Botha-Brink & Smith^19^. The individual that is used for comparison here is the second individual belonging to this specimen number, since it, in contrast to the individual considered by Botha-Brink & Smith^19^, preserves both the skull and a femur. Because the skull length of this individual of NMQR 3763 is known, it is possible to compare its size to the largest known specimen established by Botha-Brink & Smith^19^, BP/1/471. Using femur length as a proxy for size indicates that UWBM 95529 is 107.05% larger than BP/1/471 and using the midshaft width of the femur as a size proxy shows that UWBM 95529 is 147.37% larger (Table 2). The former can be considered a conservative and the latter a liberal estimate of relative size, and the newest Antarctic specimen therefore represents the largest known *Prolacerta* specimen to date it.

**Table 2:** Comparison of measurements of UWBM 95529 with two other *Prolacerta* specimens to determine the percentage of overall size compared to the hitherto largest known *Prolacerta* specimen.

specimen number	skull length (in mm)	femur length (in mm)	femur midshaft width (in mm)	% compared to the largest known specimen
BP/1/471	70.30	x	x	100
NMQR 3763	59.24	57	6.86	84.27
UWBM 95529	x	72.41	x	107.05
UWBM 95529	x	x	10.11	147.37

Measurements of NMQR 3763 were provided by Jennifer Botha-Brink.

The larger Antarctic specimen UWBM 95529 provides an excellent opportunity to test the consistency of the differences that Colbert^1^ identified between the Antarctic and South African specimens. These differences are reviewed here and discussed in the context of the new description of UWBM 95529.

**Figure 9:**
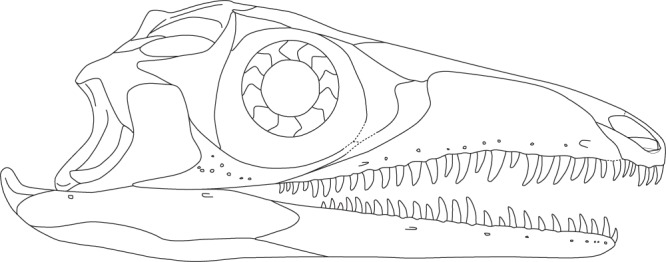
The reconstruction of the skull and lower jaw of *Prolacerta broomi*. This reconstruction is a modification of the reconstruction provided by Ezcurra^10^. The outline of the premaxilla was taken from AMNH 9502. The changes to the antorbital region where primarily based on BP/1/5375. BP/1/5880 was used for the new interpretation of the sutures of the lower jaw.

The skull morphology of *Prolacerta* from Antarctica is mainly known from one specimen (AMNH 9520, Fig. 8), in addition to a few poorly preserved and fragmentary specimens. For this study, these specimens were compared to the *Prolacerta* skulls from South Africa based on first hand observation of the specimens housed in the BP, SAM-PK, and UCMP collections. Based on the result of this comparison, an updated reconstruction of the skull of *Prolacerta broomi* is also provided here (Fig. 9). The snout region of AMNH 9520 is short relative to the region posterior to the anterior margin of the orbit compared to the South African specimens (Figs 8 and 9). Since only a few isolated skull bones are preserved, this character cannot be observed in UWBM 95529. Additionally, Colbert reconstructed four fewer marginal teeth for the Antarctic *Prolacerta* based on AMNH 9520 and AMNH 9521 than were previously reconstructed for *Prolacerta* and observed that these are in some cases more rounded compared to the more labiolingually compressed teeth in the South African specimens^1,2^. Although the former character is unobservable because not all of the marginal teeth of UWBM 95529 are preserved, the shape of the preserved teeth can be identified (Fig. 2). These are slightly labiolingually compressed, which is more in accordance with the description of the South African specimens^2,3^ than that of the Antarctic material. However the teeth of the South African specimens are described as being slightly labiolingually compressed only. Direct comparison shows that there is very little, if any, difference between the morphology of the teeth of the *Prolacerta* specimens from South Africa (mainly based on BP/1/471) and the Antarctic specimens (observed in AMNH 9520 and AMNH 9521). Colbert considered the postorbital of AMNH 9560 to be more robust than in the South African specimens. The only other postorbital hitherto known from an Antarctic specimen (preserved in AMNH 9520), is too poorly preserved for comparison. In UWBM 95529, the bone tentatively identified as the postorbital (Fig. 2), is more similar in shape to the South African specimens (e.g. BP/1/471) than the postorbital in AMNH 9560. Provided that the identification of the postorbital in UWBM 95529 is correct, the more robust morphology observed in the latter specimen is therefore not a trait specific to the Antarctic specimens. Instead, its deviating morphology could be attributable to ontogenetic variation, especially given to the very small size of AMNH 9560, in which the postorbital is only 4 mm in height.

**Figure 10:**
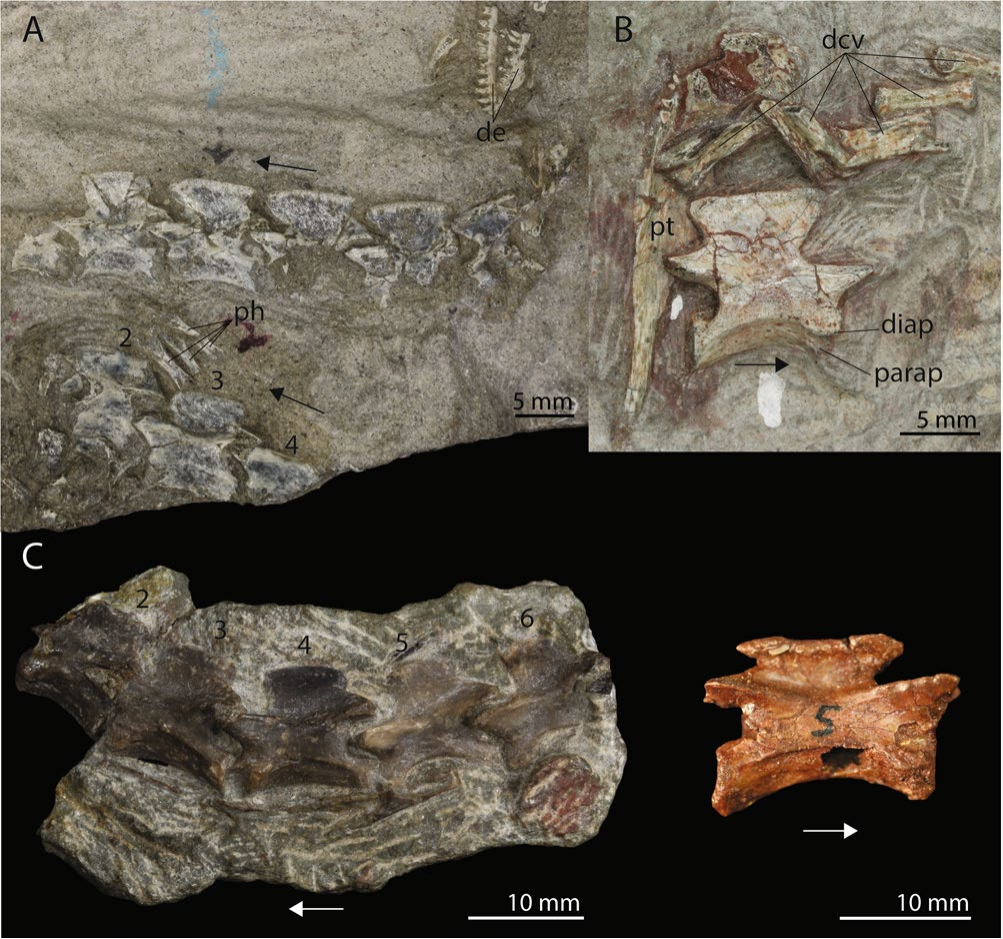
Cervical vertebrae of several specimens of *Prolacerta broomi*. (**A**) Two incomplete columns of cervical vertebrae of the Antarctic specimen AMNH 9558. (**B**) An isolated cervical vertebra of the Antarctic specimen AMNH 9568. (**C**) An incomplete column of cervical vertebrae of the South African specimen UCMP 37151. (**D**) An isolated fifth cervical vertebra of the South African specimen BP/1/2675. The arrows indicate the anterior direction. Abbreviations used in the figure are distal caudal vertebra (dcv), dentary (de), diapophysis (diap), parapophysis (parap), phalanx (ph), and pterygoid (pt).

Regarding the axial skeleton Colbert observed that the neural spines of the cervical vertebrae of the Antarctic specimens are somewhat taller and longer than those from South Africa^1^. This character unfortunately cannot be observed in UWBM 95529 since no cervical vertebrae are preserved. However, a comparison between the cervicals from South African (UCMP 37151 and BP/1/2675) and Antarctic (AMNH 9558 and AMNH 9568) specimens shows that there is also clear variation between the South African specimens. The neural spines of BP/1/2675 are shorter compared to the other specimens and the neural spines of UCMP 37151 are not expanded anteroposteriorly whereas they are in the other specimens (Fig. 10). However, no character distinguishes the specimens from the different continents. On the dorsal vertebrae Colbert commented that the neural spines are tilted somewhat posteriorly and that their centra are “pinched”, resulting in a ventral ridge. He did not specify in which specimens he observed these posteriorly tilted neural spines, but dorsal vertebrae are preserved in specimens AMNH 9535, AMNH 9564, AMNH 9519, and AMNH 9574 among the Antarctic specimens excluding UWBM 95529. They are likely also preserved in AMNH 9561; however this specimen was unfortunately not available for study. The presacral vertebrae of AMNH 9519 show strong similarities both to the cervical vertebrae of *Procolophon* as well as the dorsal vertebrae of *Prolacerta*. The neural spines of these vertebrae are mainly dorsally orientated. However, since the identity of this part of the specimen is uncertain, it is not considered here. The vertebrae in AMNH 9535 are too poorly preserved to observe the neural spines sufficiently. Therefore AMNH 9564 and AMNH 9574 are the only specimens that Colbert described in which the neural spines of the dorsal vertebrae are clearly visible. The neural spines of both of these specimens are strongly slanting posteriorly. In contrast, the neural spines of the dorsal vertebrae of UWBM 95529 are nearly fully vertically orientated. The morphology of UWBM 95529 regarding this character is therefore more similar to that seen in the dorsal vertebrae of the South African BP/1/2676 than that of the Antarctic AMNH 9564 and AMNH 9574 (Fig. 4). This makes the slanting of the dorsal neural spines a variable character for the Antarctic specimens of *Prolacerta* that could very well be dependent on ontogenetic stage.

**Figure 11:**
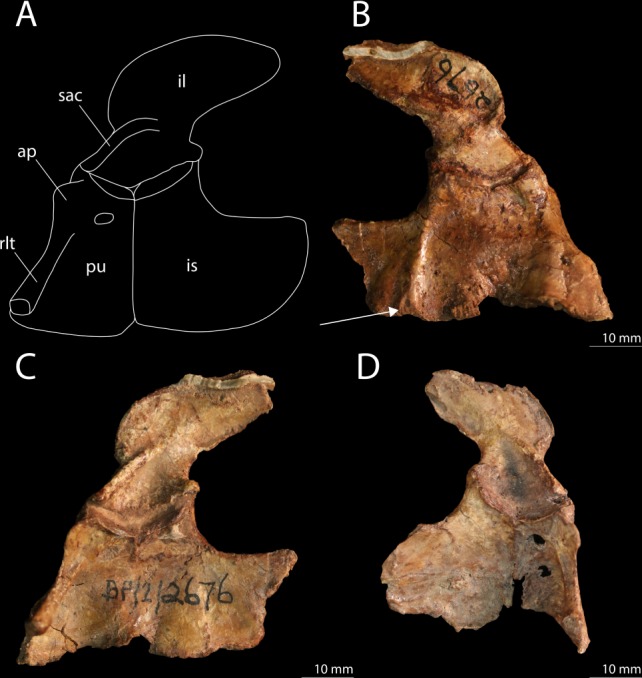
The pelvis of *Prolacerta broomi*. (**A**) A reconstruction of the pelvis in left lateral view. (**B**) The left hemipelvis of BP/1/2676 in medial view. The arrow indicates the triangular raised peduncle of the ischium. (**C**) The left hemipelvis of BP/1/2676 in lateral view. (**D**) The right hemipelvis of BP/1/2676 in lateral view. Abbreviations used in the figure are ambiens process (ap), ilium (il), ischium (is), pubis (pu), rod-like tubercle (rlt), and supraacetabular crest (sac).

Colbert used AMNH 9502 for his comparison of the pelvic girdle and hind limb between the Antarctic and South African material, in which he found a number of differences^1^. He described the ilium of AMNH 9502 as differing from South African specimens in being somewhat taller and having a straight anterior margin. It has to be pointed out that the ilium of AMNH 9502 is only partially preserved and therefore is more difficult to interpret than some of the South African specimens (most notably BP/1/2676, Fig. 11), and UWBM 95529 (Figs 5). I was not able to corroborate Colbert’s inferences from the ilium of AMNH 9502, since only the dorsalmost part of the anterior margin of the iliac blade can be seen and this does not allow for any estimate on the height of the entire bone. The ilium of UWBM 95529, in contrast, is particularly well preserved, and is generally similar to BP/1/2676. It seems that the postacetabular process is somewhat anteroposteriorly shorter in UWBM 95529, however it is unclear whether the process is completely intact posteriorly (Figs 5 and 11). As Colbert pointed out, the tibia and femur are subequal in length in AMNH 9502, whereas in the South African specimen BP/1/2676, on which Gow based his description of the hind limb of *Prolacerta*, the tibia is slightly longer than the femur. In UWBM 95529 the tibia and femur are subequal in length, as in the other Antarctic specimen AMNH 9502 and in contrast to BP/1/2676. However this difference is minor, since the tibia length of BP/1/2676 is 69.77 mm and that of the femur 64.07 mm, creating a limb ratio of 1.09, which differs little from that of UWBM 95529 (0.96, taken from measurements in Table 1). Limb proportions have been used to differentiate between three different species of the same genus in the non-archosauriform archosauromorph “protorosaur” *Macrocnemus*, namely *Macrocnemus bassanii*, *Macrocnemus fuyuanensis*, and *Macrocnemus obristi*^21,49,50^. However, the limb proportions that distinguish these species are much larger than the proportions observed here for *Prolacerta*. Additionally, although the species of *Macrocnemus* are still considered valid, it has also been suggested that these differences in limb proportions might be related to sexual dimorphism^21^. Regarding the foot, Colbert pointed out that the centrale is absent in AMNH 9502 in contrast to Gow’s reconstruction of the pes for *Prolacerta*, but Colbert argued that this bone had likely been lost during preservation. In UWBM 95529 a centrale is preserved, excluding the possibility that the Antarctic *Prolacerta* material differs from the South African *Prolacerta* in lacking a centrale (Fig. 6). Finally, Colbert directed special attention to the shape of metatarsal V, which is not hook-shaped as in most other “protorosaurian” archosauromorphs and which in contrast to the South African specimens has a strong proximal articulation in AMNH 9502. The metatarsal V of UWBM 95529 is also not very hook-shaped and its medial margin only curves comparatively gradually proximally (Fig. 6). However, the overall shape of the metatarsal V in UWBM 95529 is more similar to the one observed in BP/1/2676 than to the one in AMNH 9502. The difference that Colbert described between the metatarsal V of AMNH 9502 with that reconstructed by Gow based on BP/1/2676 is unclear, since the proximal surface of the metatarsal in AMNH 9502 does not appear to be distinctly broader. Instead, it would be more precise to point out that the medial margin is less curved, not only less so than in other “protorosaurs”, but also less than in other specimens of *Prolacerta* (UWBM 95529, BP/1/2676, and SAM-PK-7701). This observed difference is most likely dependent on the angle under which the metatarsal V was preserved in AMNH 9502, since the bone is flattened and likely not preserved exactly in dorsal view.

To summarize, no consistent differences can be observed between the Antarctic and the South African material referred to *Prolacerta broomi*. At the same time, although the postcranial morphology of the Antarctic *Prolacerta* is now better understood, the cranial morphology is not, since well-preserved adult skull material is lacking.

### New observations on the osteology of *Prolacerta*

The detailed re-evaluation of the *Prolacerta* material undertaken for this study has resulted in several new insights into *Prolacerta* morphology, which are presented and discussed here.

**Figure 12:**
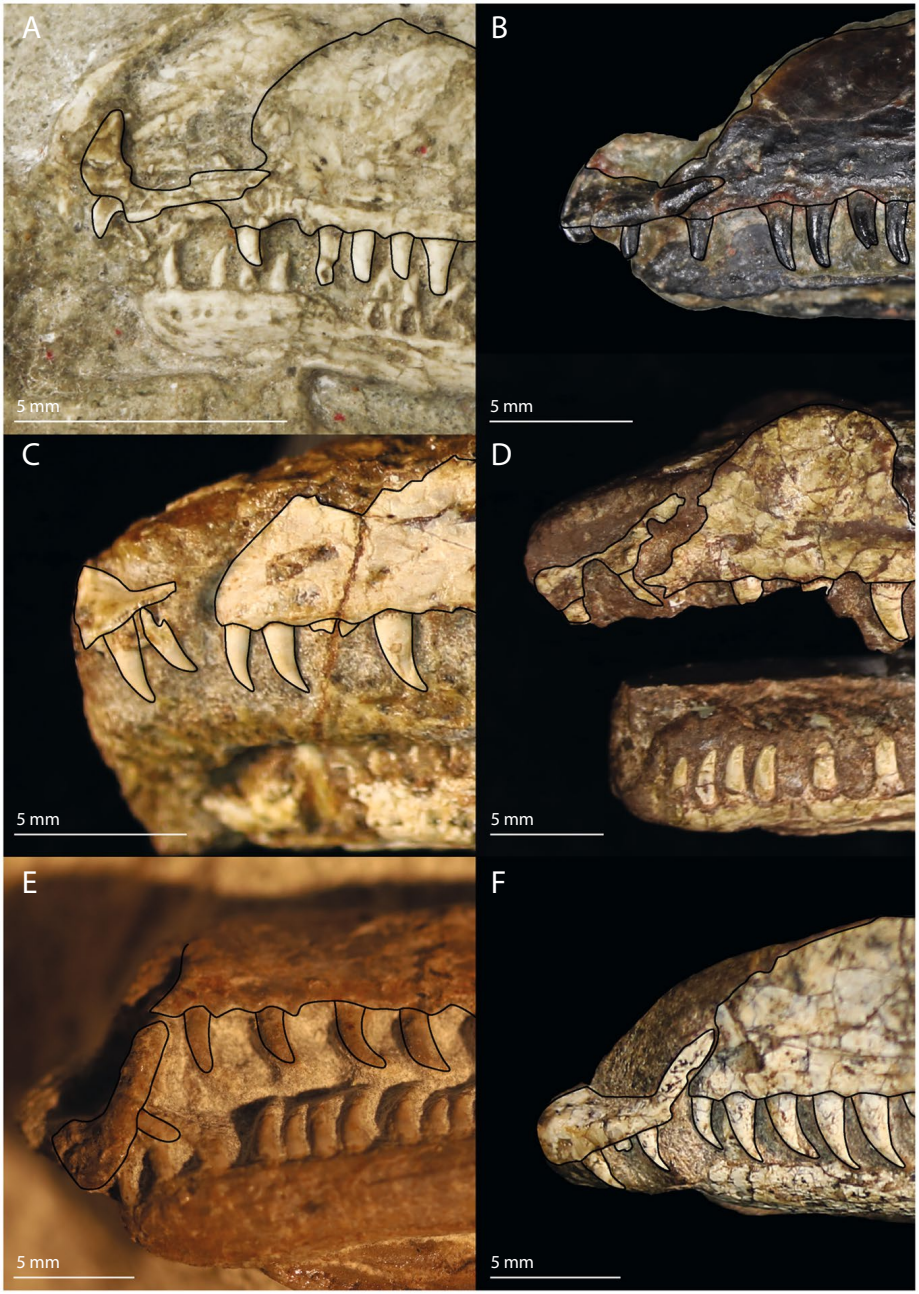
The anterior snouts of various *Prolacerta* specimens in lateral view preserving the premaxilla and maxilla. (**A**) AMNH 9520, (**B**) UCMP 37151, (**C**) BP/1/4504a (mirrored), (**D**) BP/1/471, (**E**) SAM-PK-K10802 (mirrored), (**F**) BP/1/5088.

**Figure 13:**
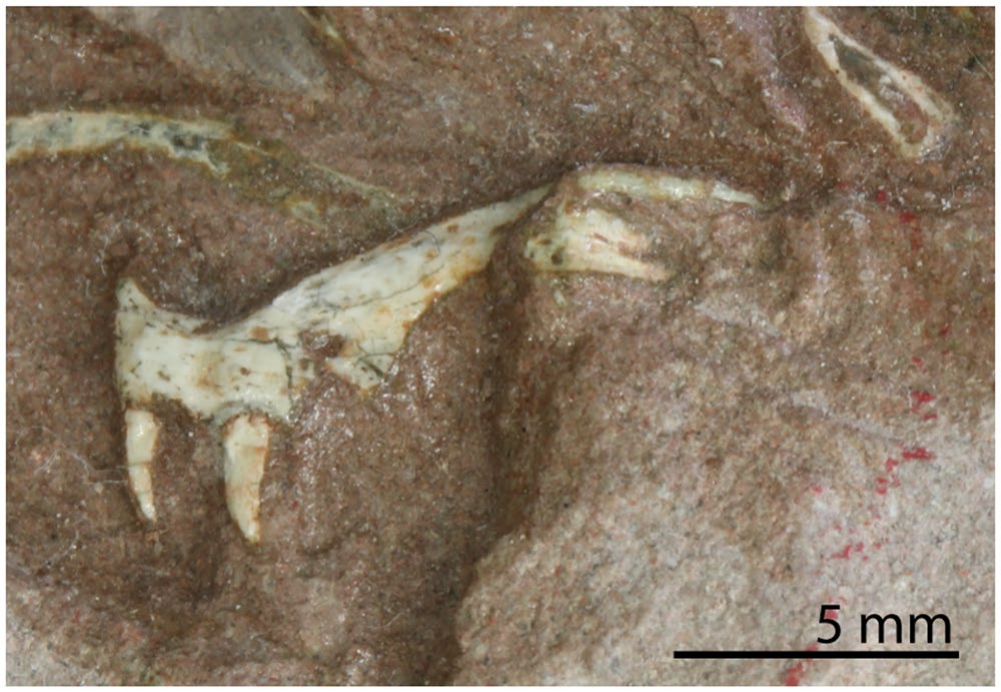
The isolated left premaxilla of AMNH 9502.

The morphology of the premaxilla and its articulation with the maxilla is an aspect of *Prolacerta* morphology that has been interpreted markedly different by various authors and in recent phylogenetic analyses. Both Gow and Benton reported a downturned premaxilla for *Prolacerta*, similar in its morphology to that of the archosauriform *Proterosuchus*^2,51^. On the other hand, Dilkes considered the ventral margin of the premaxilla of *Prolacerta* to be straight^29^. Modesto & Sues discussed the premaxilla in detail and concluded that it was downturned, albeit distinctly less so than in *Proterosuchus*^3^. This interpretation was mainly based on the right premaxilla of BP/1/471, which they described as being fully articulated with the maxilla. Unfortunately this region of the specimen has been lost and thus no first-hand observations could be made on this side of the specimen for the premaxilla (personal obervation). Ezcurra corroborated the presence of a downturned premaxilla and additionally described that it is accompanied by a notch on the ventral margin of the premaxilla-maxilla suture (Ezcurra, character 24 and 29), much as in *Proterosuchus fergusi*, in which the downturned premaxilla is a strongly pronounced and diagnostic character^10^. Ezcurra and Butler argued that this character shows a similar morphology in adult *Prolacerta* and juvenile *Proterosuchus fergusi* and that this character gets more pronounced as ontogeny progresses in *Proterosuchus*, with a very conspicuous downturned premaxilla in the adult^52^. *Prolacerta* is considered to be very closely related to Archosauriformes of which proterosuchids are some of the earliest members and therefore they hypothesized that this character might have evolved through a peramorphic shift in development and that this and other heterochronic shifts might have been important drivers of skull evolution in early Archosauriformes. In contrast, Pritchard *et al*. interpreted the margin of the premaxilla to be confluent with that of the maxilla in their recent character matrix (character 2)^13^. The premaxilla-maxilla suture is preserved in AMNH 9520, UCMP 37151, BP/1/471, BP/1/4504a, BP/1/5880, and SAM-PK-K10802 (Fig. 12). The degree to which the tooth margin of the premaxilla is downturned compared to the maxillary margin in these specimens is variable due to taphonomic distortion, being straight or nearly straight in AMNH 9520 and UCMP 37151 and downturned in BP/1/471, BP/1/4504a, BP/1/5880, and SAM-PK-K10802. With the possible exception of the now lost right premaxilla of BP/1/471, none of the *Prolacerta* specimens preserve a premaxilla in full articulation with the maxilla. This seems to indicate that the suture of the premaxilla and maxilla was comparatively weak, resulting in the premaxilla partially detaching from the maxilla and shifting from its original position and orientation after death. An indication as to the natural orientation of the premaxilla might be found in the morphology of the postnarial process, which forms the suture with the maxilla. This process is either too poorly preserved or not preserved at all to be considered in BP/1/4504a and SAM-PK-K10802. In AMNH 9520, UCMP 37151, and BP/1/471 it is preserved and slightly dorsally facing but largely confluent with the tooth margin of the premaxilla. In BP/1/5880 however it is more dorsally orientated, which might represent a taphonomic artifact. A well-preserved isolated premaxilla is preserved in AMNH 9502 (Fig. 13). In this specimen the postnarial process is slightly dorsally oriented compared to the tooth-bearing margin. A similar displacement of the premaxilla and morphology of the postnarial process can be observed in various specimens of *Macrocnemus*^21^. For this taxon it was suggested that the sutural contact between the premaxilla and maxilla suture could represent a true morphological feature, but that it cannot be excluded that it represents a taphonomic artifact. In contrast to *Prolacerta* and *Macrocnemus*, the postnarial process of *Proterosuchus* is somewhat ventrally tilted from the premaxillary tooth margin. The similarity in morphology of this region in *Macrocnemus* and *Prolacerta* is relevant to the hypothesis of Ezcurra and Butler^52^, since *Macrocnemus* is much less closely related to *Proterosuchus* than *Prolacerta* is, indicating that this feature is more common in early archosauromorphs than previously thought. In addition, *Macrocnemus* is known from the Anisian-Ladinian (Middle Triassic), whereas *Prolacerta* and *Proterosuchus* are both known from the Induan (Early Triassic). Furthermore, *Teyujagua paradoxa*, an early archosauromorph from the Early Triassic of Brazil, possesses a slightly downturned premaxilla, but lacks the notch on the ventral margin of the premaxilla-maxilla suture^14^. In addition, like *Proterosuchus* but unlike *Prolacerta*, the postnarial process of *Teyujagua* is somewhat ventrally orientated. *Teyujagua* was recovered as the closest sister taxon of Archosauriformes, and is thus considered to be more closely related to proterosuchids than *Prolacerta*. A direct transition in the morphology of the premaxilla from *Prolacerta* to *Proterosuchus* through a peramorphic shift as proposed by Ezcurra and Butler^52^ therefore seems unlikely. A more likely scenario is that the presence of a downturned premaxilla first evolved in a common ancestor of *Teyujagua* and Archosauriformes, before becoming a very strongly displayed character for proterosuchids. Thus, the morphology seen in *Prolacerta* does not necessarily constitute an onset towards the strongly downturned premaxilla of *Proterosuchus* and as in *Macrocnemus* it remains somewhat unclear whether there was a notch on the ventral margin of the premaxilla-maxilla suture and whether the premaxilla was slightly downturned in *Prolacerta*.

Other new insights into the skull and lower jaw morphology of *Prolacerta* relate to the region directly anterior to the orbit and the sutures between the dentary, surangular, and angular. These changes were incorporated into the new reconstruction of the skull and lower jaws, which represents a modification of the one provided by Ezcurra (Fig. 9)^10^. These modifications are the following. The septomaxilla can now be observed through the external naris. The morphology of the premaxilla and its articulation with the maxilla has been modified according to the findings discussed above. The posterior end of the dorsal margin of the maxilla is more abrupt and consequently the dorsal portion of the lacrimal is distinctly wider. In addition the posterior portion of the nasals expands laterally and the anterior margin of the prefrontal extends less anteriorly and is curved. These changes have been made mainly based on BP/1/5375 in which this region is preserved best among *Prolacerta* specimens. The dentary bears two posterior processes instead of one, with the first on the dorsal end of the bone and the second more or less located at mid-height on the posterior margin of the dentary. Finally, the surangular-angular suture is slightly adjusted. The main reference for these changes is BP/1/5880 in which these bones and their sutures are the clearest.

Although *Prolacerta* is one of the best represented early archosauromorph taxa, its postcranial morphology remains poorly known since almost all studies have focused on cranial anatomy^3,6,9,20^, with the postcranial anatomy only being discussed by Gow and Colbert^1,2^. The focus on cranial anatomy can be attributed to the role *Prolacerta* has played in discussions of early archosauromorph relationships which have mainly relied on cranial characters as well as very few articulated *Prolacerta* skeletons being known historically in comparison to skulls. Gow described the postcranium of *Prolacerta* based on two, very well-preserved specimens, BP/1/2675 and BP/1/2676, that due to acid preparation are largely disarticulated. Other *Prolacerta* specimens with postcranial remains are present at the SAM-PK and NMQR (e.g. SAM-PK-K10802, SAM-PK-K10620, and NMQR 3763) but have not been described osteologically. They are referred to for comparison here, but a full osteological description of this material is beyond the scope of this study.

Gow hypothesized *Prolacerta* to have fed on small tetrapods as well as insects and the tooth morphology of *Prolacerta* is consistent with those of a predator^2^. UWBM 95529 is the first *Prolacerta* specimen to preserve stomach contents and confirms that *Prolacerta* fed on small vertebrates (Fig. 6). Furthermore, the fact that the vertebrae of the prey item are still in articulation seems to indicate that *Prolacerta* swallowed its prey whole, much like many modern saurians.

No intercentra are preserved in UWBM 95529 (Fig. 4). For *Prolacerta*, postaxial intercentra can be observed in BP/1/2676, BP/1/2675, and UCMP 37151. UCMP 37151 preserves cervical postaxial intercentra and no other vertebrae are preserved. In BP/1/2675, in which the postcranium is almost fully disarticulated, the only postaxial vertebra with a preserved intercentrum is a mid-dorsal. In BP/1/2676 a large part of the vertebral column is preserved, and postaxial intercentra can be observed for both the cervical and dorsal vertebrae. It appears that the presence or absence of intercentra in *Prolacerta* is variable since the vertebral column of UWBM 95529 is very well-preserved and articulated, meaning the intercentra do not appear to have been lost during preservation, yet no intercentra are present.

**Figure 14:**
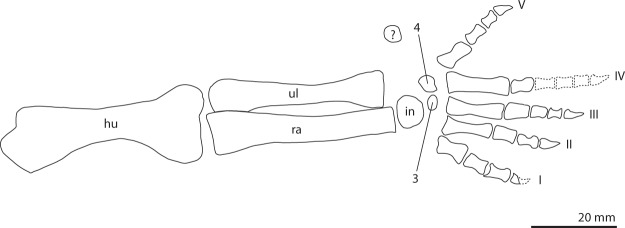
A reconstruction of the right forelimb of *Prolacerta broomi* in ventral view based of UWBM 95529. The left humerus was used in the reconstruction because more morphology was visible on this element than on the equivalent of the right forelimb. All other elements were taken from the right forelimb. Abbreviations used in the figure are intermedium (in), humerus (hu), radius (ra), and ulna (ul). The Western Arabic numerals indicate the number of each distal carpal and the Roman numerals the number of each digit.

The manus in UWBM 95529 is largely articulated and a phalangeal formula of 3-3-4-?-3 can be established (Fig. 7). This contradicts with the formula of 2-3-4-5-3 provided by Gow^2^. This discrepancy might be explained by the fact that Gow based his reconstruction on the manus of BP/1/2675, which is fully disarticulated. Thus, assuming UWBM 95529 does not belong to a different taxon with a different manual formula, the combined evidence from UWBM 95529 and BP/1/2675 indicates that the manual phalangeal count for *Prolacerta* is 3-3-4-5-3, although a formula of 3-3-4-4-3 cannot be excluded (Fig. 14).

The pelvic girdle of *Prolacerta* was described by Gow based on BP/1/2676 in which both sides of the pelvic girdle are completely preserved (Fig. 11)^2^. A nearly fully complete and articulated right hemipelvis visible in medial view is also preserved in SAM-PK-10802. A comparison of these specimens with the pelvic elements preserved in UWBM 95529 has resulted in new observations, which merit a new reconstruction of the pelvic girdle of *Prolacerta* (Fig. 11A). A clear tuberous projection can be seen on the proximal anterior margin of the pubis. This constitutes the ambiens process which is present in the majority of early archosauromorphs^10^. However, this element was previously not reported for *Prolacerta* by any author. Furthermore, the reconstruction by Gow shows a ventral margin of the ischium which is based on the morphology of these elements in BP/1/2676^2^. In this specimen the posteriormost point of the ischium is distinguished from the rest of the ischial blade by a concavity on the ventral margin of ischium, creating a distinct posterior process (Fig. 11). However, the margins seem to be partially broken in this specimen. In UWBM95529, except for the anteriormost part, the entire ventral margin is visible and intact. Here no posterior process is visible, but instead the ventral margin of the ischium is curved continuously from its posteriormost point (Fig. 5). Finally, although somewhat smaller in size, a triangular raised platform or peduncle on the medial side of the ischium, similar to the pronounced articulation surface on the ischial margin described for allokotosaurs^12^, can be observed on the ischia of UWBM 95529 and BP/1/2676 (Figs 5 and 11B).

### Phylogenetic implications

**Figure 15:**
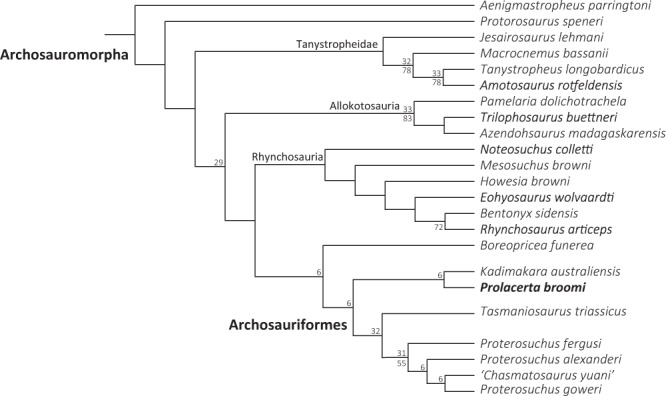
Strict consensus tree of 75 MPTS (2647 steps, C.I: 0.296, R.I: 0.618) of early archosauromorphs showing all the non-archosauriform archosauromorphs and proterosuchids. Bremer values above 1 and Bootstrap frequencies above 50% are provided above and below each node, respectively.

To test the implications of the osteological revision of *Prolacerta*, the relevant characters were altered in the matrix of Ezcurra^10^, which represents the most recent and extensive phylogenetic dataset of early archosauromorphs including *Prolacerta* and most of its closest relatives. The resultant tree shows the same topology as the original tree by Ezcurra^10^ (Fig. 15). The analysis recovered 75 most parsimonious trees (MPTs) with a length 2647 steps, in contrast to the 40 most parsimonious trees with 2646 steps each recovered by Ezcurra^10^.

### Biogeographical implications

Since the first description of tetrapod fossils from the Fremouw Formation of Antarctica, which has a temporal range from the latest Permian to Middle Triassic^53^, the similarities of the faunal composition between this locality and the Permo-Triassic AZs of the South African Karroo Basin have been recognized^15,16^. Since then many new findings from these localities have provided a more detailed picture of this biogeographical issue. It was previously showed that endemism increased during the Early and particularly the Middle Triassic in southern Pangaea following the End-Permian Mass Extinction Event, but that the Antarctic fauna differentiated less from the South African fauna than other South Pangaean basins in Malawi, Tanzania, and Zambia^18^. Recently, archosauromorph remains from the lower Fremouw Formation were described with a reconstructed total body length of between 2.0 and 2.4 metres that were different and larger in size than any of the Early Triassic archosauromorphs from the well-sampled *Lystrosaurus* AZ of South Africa, indicating a possibly higher maximally attained body size for the Antarctic fauna^5^. As was previously suggested, the presence of larger tetrapods in Antarctica could represent an example of Bergmann’s rule, which states that individuals of the same taxon or very closely related taxa attain larger body sizes at higher latitudes^54^. Although more specimens of various taxa are required for a detailed investigation, the large size of UWBM 95529 compared to the South African *Prolacerta* specimens does provide one line of evidence that could further support this suggestion. Apart from the apparently larger maximum size, the Antarctic material cannot be morphologically distinguished from the South African specimens of *Prolacerta broomi*. This further supports the already well established close association of the tetrapod faunas between the lower Fremouw Formation and *Lystrosaurus* AZ of South Africa.

## Conclusions

A newly discovered Antarctic specimen of *Prolacerta broomi* (UWBM 95529), which represents the largest specimen and most complete articulated skeleton of the species, is presented. Its morphology, together with that of previously described Antarctic specimens that were previously described by Colbert^1^, is compared to Prolacerta broomi specimens from South Africa. No clear morphological differences are present between the specimens from these two regions and therefore UWBM 95529 is identified here as *Prolacerta broomi*. In addition, the identification of the other Antarctic *Prolacerta* specimens to *Prolacerta broomi* by Colbert is corroborated. The large size of UWBM 95529 supports the hypothesis that Bergmann’s rule potentially applies to the Triassic tetrapod faunas of southern Pangaea.

The discovery of UWBM 95529 has led to several new insights into the lifestyle and morphology of *Prolacerta*. Its stomach contents confirm that *Prolacerta* preyed on small vertebrates and the maximum size of *Prolacerta* is expanded. The phalangeal formula of the manus is modified to 3-3-4-4/5-3. First hand re-evaluation of the majority of *Prolacerta* specimens reveals that the morphology of the premaxilla and its suture with the maxilla is more similar to the morphology of the tanystropheid *Macrocnemus* than to that of the archosauriform *Proterosuchus* as previously reported. The connection between the maxilla and premaxilla was likely loose and the orientation of the premaxilla to the maxilla was straight or very slightly downturned, rather than distinctly downturned with a notch on the ventral margin of the premaxilla-maxilla suture. Additional new findings regarding the morphology of the skull and lower jaw, as well as the postcranial morphology of *Prolacerta* is revised and discussed. New reconstructions for the skull and lower jaw, the forelimb, and pelvic girdle are presented. A new phylogenetic analysis including these new findings is performed, which yields no topological changes compared to the previous analysis.

## Methods

### Phylogenetic analysis

The character matrix of Ezcurra^10^ was used with the scorings for *Prolacerta* modified according to the findings of this study. The 81 included taxa (OTUs) correspond to the taxa of analysis 3 (reduced analysis) of Ezcurra^10^, as they exclude *nomina dubia* and taxa with large amounts of missing data. Out of the 600 characters, the scoring of the following was altered for *Prolacerta*: character 29 from 1 to ?; character 24 from 1 to ?; character 58 from 0 to 1; character 273 from 0 to 1; character 366 from 0 to 0/1; character 468 from 1 to 0; and character 474 from 1 to 0. *Petrolacosaurus kansensis* was set as outgroup. Maximum parsimony analyses were performed using the ‘New Technology Search’ algorithms of TNT version 1.5^55^. First, initial trees were calculated using the ‘Sectorial Search’, ‘Ratchet’, ‘Drift’ and ‘Tree Fusing’ algorithms, with random addition sequences (RAS) set to 1000, keeping all trees found, and 100 iterations for each mentioned algorithm. The number of suboptimal trees to be retained was set at 10 and the relative fit difference at 0.1. The resulting initial trees were subsequently subjected to two separate sets of analyses of three rounds each. The one set of analyses consisted of a first round of 1000 iterations of ‘Sectorial search’, followed by a second round of 1000 iterations of ‘Ratchet’, and a third round of again 1000 iterations of ‘Sectorial search’. The other set comprised a first round of 1000 iterations of ‘Ratchet’, a second of 1000 iterations of ‘Sectorial Search’, and a third of 1000 iterations of ‘Ratchet’. All rounds of both sets additionally included 1000 iterations of ‘Tree fusing’. The resulting trees of both sets were combined for the calculation of the strict consensus tree, after discarding all suboptimal trees. Bootstrap supports were estimated using a ‘Traditional Search’ of 1000 iterations. Bremer support values and consistency and retention indices were calculated using the TNT scripts Bremer.run and Stats.run.

## Electronic supplementary material

Supplementary Information
